# Osteoclast‐Derived SLIT3 Mediates Osteoarthritis Pain and Degenerative Changes

**DOI:** 10.1002/advs.202517545

**Published:** 2025-11-19

**Authors:** Weiwei Zhu, Wenpin Qin, Jialu Gao, Yihan Guo, Xiaoxiao Han, Zhangyu Ma, Xiaokang Zhang, Jie He, Jing Liu, Bo Gao, Changjun Li, Lina Niu, Jianfei Yan, Kai Jiao

**Affiliations:** ^1^ Department of Stomatology Tangdu Hospital & State Key Laboratory of Oral and Maxillofacial Reconstruction and Regeneration & School of Stomatology The Fourth Military Medical University Xi'an Shaanxi 710032 China; ^2^ State Key Laboratory of Oral and Maxillofacial Reconstruction and Regeneration & National Clinical Research Center for Oral Diseases & Shaanxi Key Laboratory of Stomatology School of Stomatology The Fourth Military Medical University Xi'an Shaanxi 710032 China; ^3^ Institute of Orthopaedic Surgery Xijing Hospital Fourth Military Medical University Xi'an Shaanxi 710032 China; ^4^ Department of Endocrinology Endocrinology Research Center Xiangya Hospital of Central South University National Clinical Research Center for Geriatric Disorders Xiangya Hospital Key Laboratory of Organ Injury Aging and Regenerative Medicine of Hunan Province Changsha Hunan 410000 China; ^5^ State Key Laboratory of Oral & Maxillofacial Reconstruction and Regeneration National Clinical Research Center for Oral Diseases Shaanxi Clinical Research Center for Oral Diseases Department of Oral and Maxillofacial Surgery School of Stomatology The Fourth Military Medical University Xi'an Shaanxi 710032 China

**Keywords:** osteoarthritis, pain, sensory nerves, SLIT3, temporomandibular joint

## Abstract

Temporomandibular joint osteoarthritis (TMJ‐OA) is a prevalent degenerative joint disease that significantly impairs quality of life. Neurogenesis is considered a key initiating factor in this pain; however, the precise mechanisms remain unclear. This study tests the hypothesis that osteoclast‐derived slit guidance ligand 3 (SLIT3) plays an important role in osteoarthritis pain. These findings reveal that in TMJ‐OA mice, increased osteoclast activation and SLIT3 expression occur in the subchondral bone of the TMJ condyle, accompanied with pain. Interestingly, results from immunofluorescent co‐staining and fluorescence‐activated cell sorting support that osteoclasts serve as the primary cellular source of SLIT3 in subchondral bone, and SLIT3 produced by TRAP‐positive (TRAP^+^) osteoclasts significantly promotes the growth of sensory nerves. The results of in vivo models demonstrate that the specific knockdown/knockout of *Slit3* in TRAP^+^ osteoclasts significantly reduces the level of SLIT3. More importantly, *Slit3* knockdown/knockout in osteoclasts results in reduced sensory nerve innervation in the osteochondral regions, decreased osteoarthritis pain, and alleviated bone and cartilage degeneration in TMJ‐OA. Thus, SLIT3 derived from TRAP^+^ osteoclasts in the subchondral bone plays a crucial role in the progression of TMJ‐OA. This suggests that targeting SLIT3 might represent a promising therapeutic approach to alleviate the pain in TMJ‐OA.

## Graphical abstract

In TMJ‐OA, osteoclasts play a significant role in promoting the growth of sensory nerves at the osteochondral interface. In early OA, TRAP^+^ osteoclast‐derived SLIT3 induces sensory nerve growth into the condylar cartilage. This nerve growth facilitates the development of pain associated with OA. Additionally, this process is accompanied by a certain degree of bone and cartilage degradation.

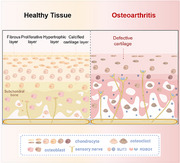

## 1 Introduction

Temporomandibular joint osteoarthritis (TMJ‐OA) is a chronic, progressive joint disease characterized by the degeneration of condylar cartilage and remodeling of subchondral bone. The main symptoms experienced by patients include joint pain, limited mandibular movement, and functional impairment, which can severely affect their physical and mental well‐being.^[^ ^1^ ^]^ Pain is typically an early manifestation of TMJ‐OA and is one of its most common symptoms.^[^ ^2^ ^]^ There are currently no effective methods to completely halt the progression of TMJ‐OA;^[^ ^3^, ^4^, ^5^, ^6^, ^7^, ^8^ ^]^ thus, alleviating pain becomes the primary treatment goal for patients with TMJ‐OA.^[^ ^9^ ^]^ Consequently, identifying the sources and mechanisms of TMJ‐OA‐associated pain is of great significance to relieve patient symptoms and improve function.

It is generally believed that osteoarthritis (OA) pain is a type of nociceptive pain, involving both the central and peripheral nervous systems.^[^ ^10^ ^]^ Acute pain typically serves as part of the body's defense mechanism.^[^ ^11^, ^12^ ^]^ If acute pain caused by peripheral injury is not controlled, it can cause unnecessary suffering, thus reducing life quality, and it can lead to chronic pain through central sensitization.^[^ ^13^ ^]^ Descending pain modulatory pathways originating from the central nervous system play a crucial role in regulating peripheral nociceptor sensitivity. The descending projections are sent from key supraspinal structures, including the periaqueductal gray matter, reticular formation, and nucleus raphe magnus. The synapse within the spinal dorsal horn exerts bidirectional control over incoming peripheral nociceptive signals through both inhibitory and facilitatory mechanisms.^[^ ^14^, ^15^ ^]^ This vicious cycle of peripheral and central pain is an important reason why OA chronic pain is difficult to treat.

Normal articular cartilage is avascular and aneural, whereas neovascularization and innervation represent hallmark features of OA cartilage. During OA progression, the infiltration of nerves and vessels into degenerated cartilage leads to the release of inflammatory mediators (including cytokines such as IL‐1β, TNF‐α, and IL‐6) from chondrocytes and elevated nerve growth factor (NGF) levels.^[^ ^16^ ^]^ Concurrently, these degenerated chondrocytes also secrete neurotransmitters that activate peripheral nerve endings, thereby initiating pain signaling cascades. Furthermore, both inflammatory mediators and neurotransmitters can diffuse across the osteochondral junction, exert nociceptive stimulation on sensory nerves in subchondral bone and ultimately contribute to pain pathogenesis.^[^ ^17^ ^]^ Therefore, lesions in the subchondral bone are thought to be the primary source of OA pain.

Developed from neuroectoderm, the temporomandibular joint (TMJ) undergoes significant peripheral nervous regulation during development.^[^ ^18^ ^]^ Anatomically, the absence of a tidemark at the osteochondral junction in the TMJ condyle facilitates direct bone‐cartilage interactions, making it an ideal model for studying their crosstalk.^[^ ^19^, ^20^ ^]^ Functionally, the close relationship between TMJ biomechanics and dental occlusion enables noninvasive establishment of mechanically induced OA models through occlusion disorder, effectively eliminating the interference of surgical trauma on the experimental results.^[^ ^21^, ^22^ ^]^ Our research has demonstrated that in the early stages of TMJ‐OA, abnormal stress disrupted homeostasis and activated the metabolic activities of osteoclasts.^[^ ^9^ ^]^ Previous research demonstrated that slit guidance ligand 3 (SLIT3) was highly expressed in osteoblasts^[^ ^23^, ^24^ ^]^; however, recent studies have shifted this perspective and identified osteoclasts—not osteoblasts—were the primary source of SLIT3.^[^ ^25^, ^26^ ^]^ In this study, by specifically intervening *Slit3* in TRAP^+^ osteoclasts using adeno‐associated viruses (AAV) and crossing the TRAP‐Cre mice with *Slit3* ^flox/flox^ mice, we demonstrated the important role of TRAP^+^ osteoclasts and SLIT3 derived in TMJ‐OA and OA pain. The results underscore the importance of understanding the molecular mechanisms underlying joint degeneration and pain, paving the way for more effective and targeted treatments.

## 2 Results

### 2.1 Increased Sensory Nerve Innervation Accompanied by Pain in TMJ‐OA

**Figure 1:**
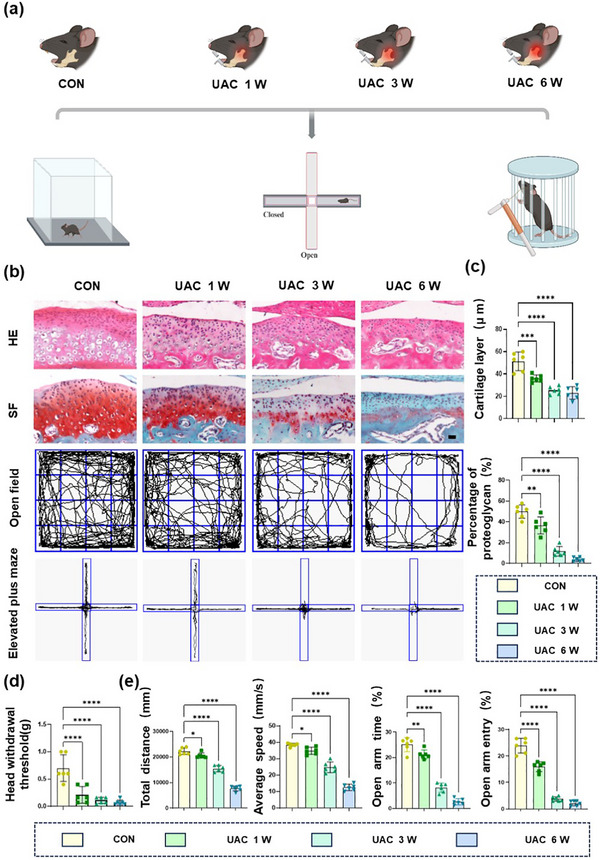
Increased osteoarthritis pain in TMJ‐OA. a) Schematic of behavior tests. b) Representative results of Hematoxylin–Eosin (HE) Staining, Safranin O (SF) staining, OFT and EPM in the CON and UAC groups. Scale bars: 70 µm. c–e) Quantitative analysis of HE staining, SF staining, Von Frey experiment, the open field test and the elevated plus maze test. *n* = 6. Scale bars: 70 µm. Statistical analyses were performed using one‐way ANOVA with Holm–Šidák multiple comparison tests. ^*^ *p* < 0.05. ^**^ *p* < 0.01. ^***^ *p* < 0.001. ^****^ *p* < 0.0001.

The previously described unilateral anterior crossbite (UAC) method was used to induce TMJ‐OA (**Figure** **1**a). The results showed that the cartilage thickness of the TMJ‐OA mice gradually decreased, and the proteoglycan content was significantly reduced as molding time increased (Figure 1b,c). Quantitative analysis of Hematoxylin–Eosin (HE) staining showed a significant increase in the relative thickness of the hypertrophic layer (hypertrophic/total layer) and the proportion of hypertrophic chondrocytes in the UAC group, despite a reduction in the layer's absolute thickness (Figure S1, Supporting Information). We have performed immunofluorescence analysis for Collagen X (Col X), a definitive molecular marker of hypertrophy.^[^ ^27^ ^]^ The new data reveal that while the hypertrophic layer of condylar cartilage is thinned in UAC groups, the relative area occupied by ColX‐positive hypertrophic chondrocytes is significantly increased, indicating an exacerbation of hypertrophy (Figure S1, Supporting Information). Studies have shown that mice experiencing pain not only exhibit behaviors related to pain, but also show signs of depression.^[^ ^28^, ^29^ ^]^ Therefore, we adopted the relevant behavioral tests to characterize the pain status of the TMJ‐OA mice, including the Von Frey test, open field test (OFT), and elevated plus maze test (EPM). The results of the Von Frey test showed that the pain threshold of the TMJ‐OA mice decreased (Figure 1d), which was associated with molding time, indicating an intensification of pain in TMJ‐OA. A reduced total distance traveled and average speed in OFT, fewer entries into the open arms and shorter durations spent there in EPM (Figure 1b,e) suggested increased pain and significant depressive behavior.

**Figure 2:**
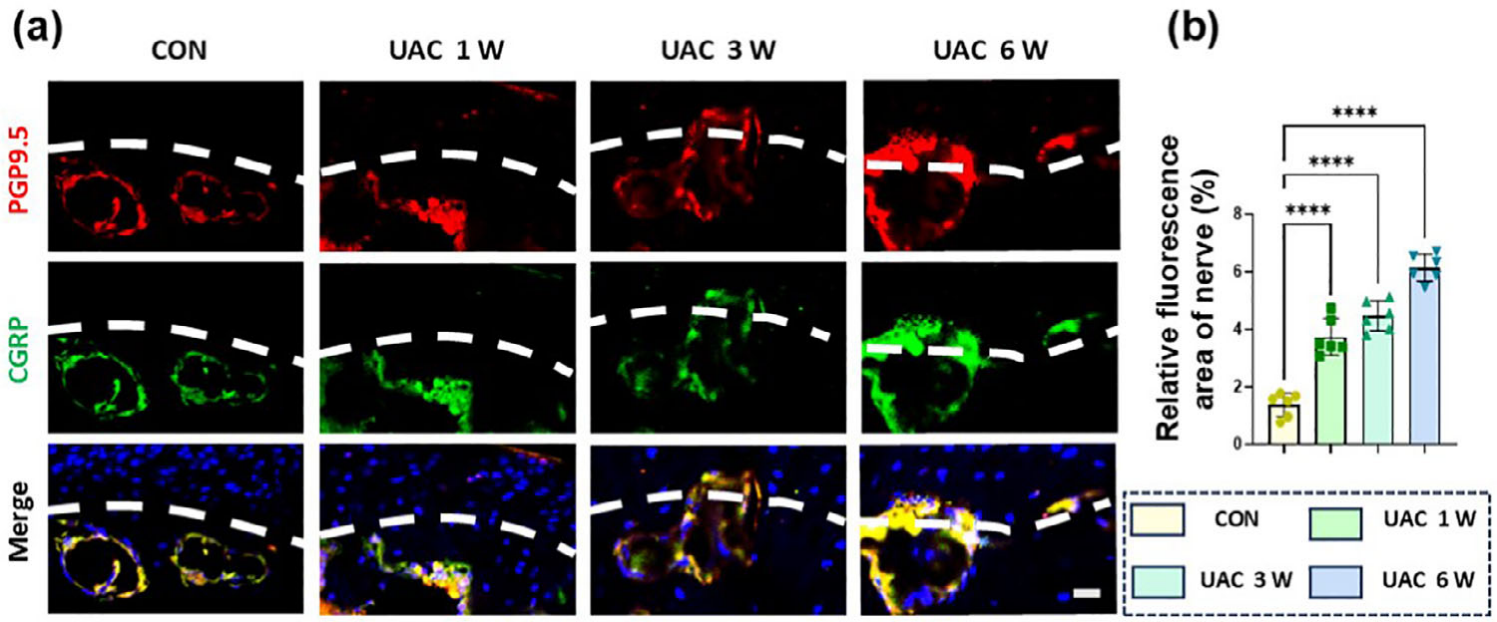
Increased subchondral sensory nerves in TMJ‐OA accompanied by pain. a) Representative images of PGP9.5 (red) and CGRP (green) co‐stained cells along the TMJ osteochondral junction in the CON and UAC groups. The white dashed line represents the boundary between bone and cartilage. Scale bars: 10 µm. b) Quantitative analysis in panel (a). *n* = 6. Statistical analyses were performed using one‐way ANOVA with Holm–Šidák multiple comparison tests. ^****^ *p* < 0.0001.

Although the mechanism of OA pain is complex, previous studies have indicated that peripheral nerve stimulation from joint tissues led to pain.^[^ ^30^ ^]^ Additionally, the increase in local sensory nerve endings in the subchondral bone is a major source of peripheral pain.^[^ ^31^, ^32^ ^]^ Therefore, in addition to observing pain manifestations, we examined the innervation of sensory nerves in the subchondral bone. Immunofluorescence staining for PGP9.5 and CGRP was performed to study the distribution of sensory nerves associated with osteogenesis at the osteochondral junction. The results showed that the subchondral trabecular bone in the control group (CON) contained a large number of nerves, while terminal branches were unable to pass through the small channels in the subchondral plate to directly contact the calcified cartilage layer (**Figure** **2**a,b). In the UAC group, the sensory nerves penetrated into noncalcified cartilage, disrupting the integrity of the osteochondral connection (Figure 2a,b). Overall, these results indicated that in mice with OA pain, the number of sensory nerves in the subchondral bone region increased. However, the specific mechanisms require further exploration.

### 2.2 Osteoclast‐Mediated TMJ‐OA Pain and Nerve Ingrowth

**Figure 3:**
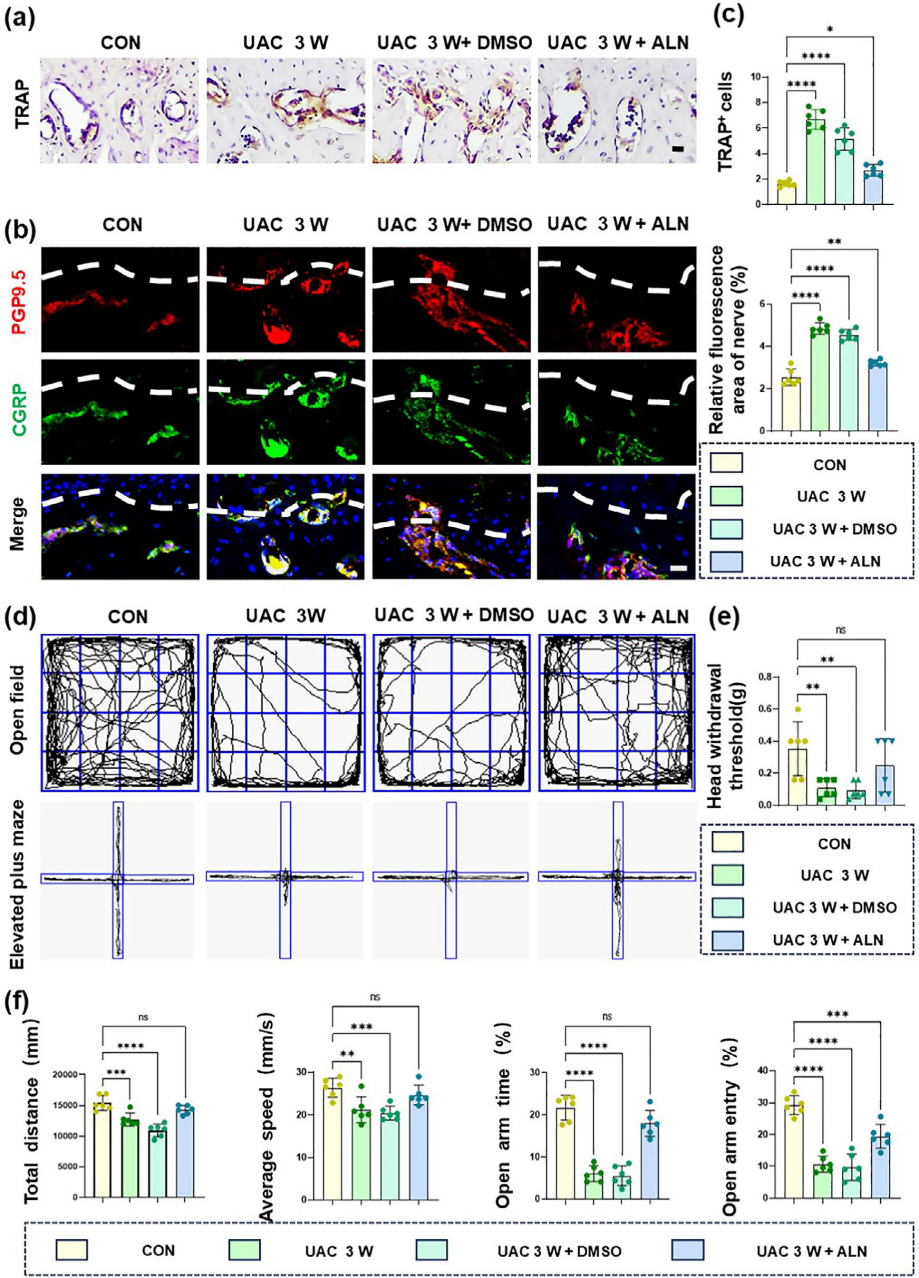
Osteoclasts mediate TMJ‐OA pain and nerve growth. a) Representative images of TRAP immunohistochemical staining in the condyle. Scale bars: 70 µm. b) Representative images of PGP9.5 (red) and CGRP (green) co‐stained cells along the TMJ osteochondral junction in the CON and UAC groups. The white dashed line represents the boundary between bone and cartilage. Scale bars: 10 µm. c) Quantitative analysis in panels (a) and (b). *n* = 6. d) Representative results of OFT and EPM in the CON and UAC groups. e) Quantitative analysis of Von Frey experiment. *n* = 6. f) Quantitative analysis of OFT and EPM. *n* = 6. Scale bars: 10 µm. Statistical analyses were performed using one‐way ANOVA with Holm–Šidák multiple comparison tests. ^*^ *p* < 0.05. ^**^ *p* < 0.01. ^***^ *p* < 0.001. ^****^ *p* < 0.0001. *ns*: no significance.

Abnormal subchondral bone remodeling plays a crucial role in the ingrowth of sensory nerves and the occurrence of peripheral pain.^[^ ^33^ ^]^ In the early progression of OA, the activity of osteoclasts significantly increases, promoting bone remodeling and leading to bone loss.^[^ ^9^ ^]^ Immunohistochemistry, TRAP staining, and immunofluorescence staining showed that compared with those in CON, the number of TRAP^+^ osteoclasts increased in the subchondral bone of the UAC 3 W mice (**Figure** **3**a,c; Figure S2, Supporting Information), along with an increase in CGRP^+^ and PGP9.5^+^ sensory nerves (Figure 3b,c). Then, alendronate sodium (ALN), a kind of bisphosphonate that inhibits osteoclasts’ function and subchondral bone remodeling,^[^ ^34^, ^35^, ^36^ ^]^ was injected intraperitoneally at a dose of 1 mg kg^−1^ 3 times per week for 3 weeks post‐UAC modeling. Treatment with ALN reduced the number of TRAP^+^ osteoclasts and sensory nerves (Figure 3a–c). Furthermore, the results of behavioral tests showed that the pain threshold of the UAC mice treated with ALN increased, indicating reduced pain (Figure 3e). The results of OFT and EPM showed that the total distance traveled and average speed in the open field, as well as the number of entries and duration in the open arms, increased in the UAC mice treated with ALN, indicating a reduction of pain and depressive behavior (Figure 3d,f). It is suggested that ALN inhibits nerve growth and OA pain through osteoclast activity.

### 2.3 Confirmation of the Effect of SLIT3 on Sensory Nerves

**Figure 4:**
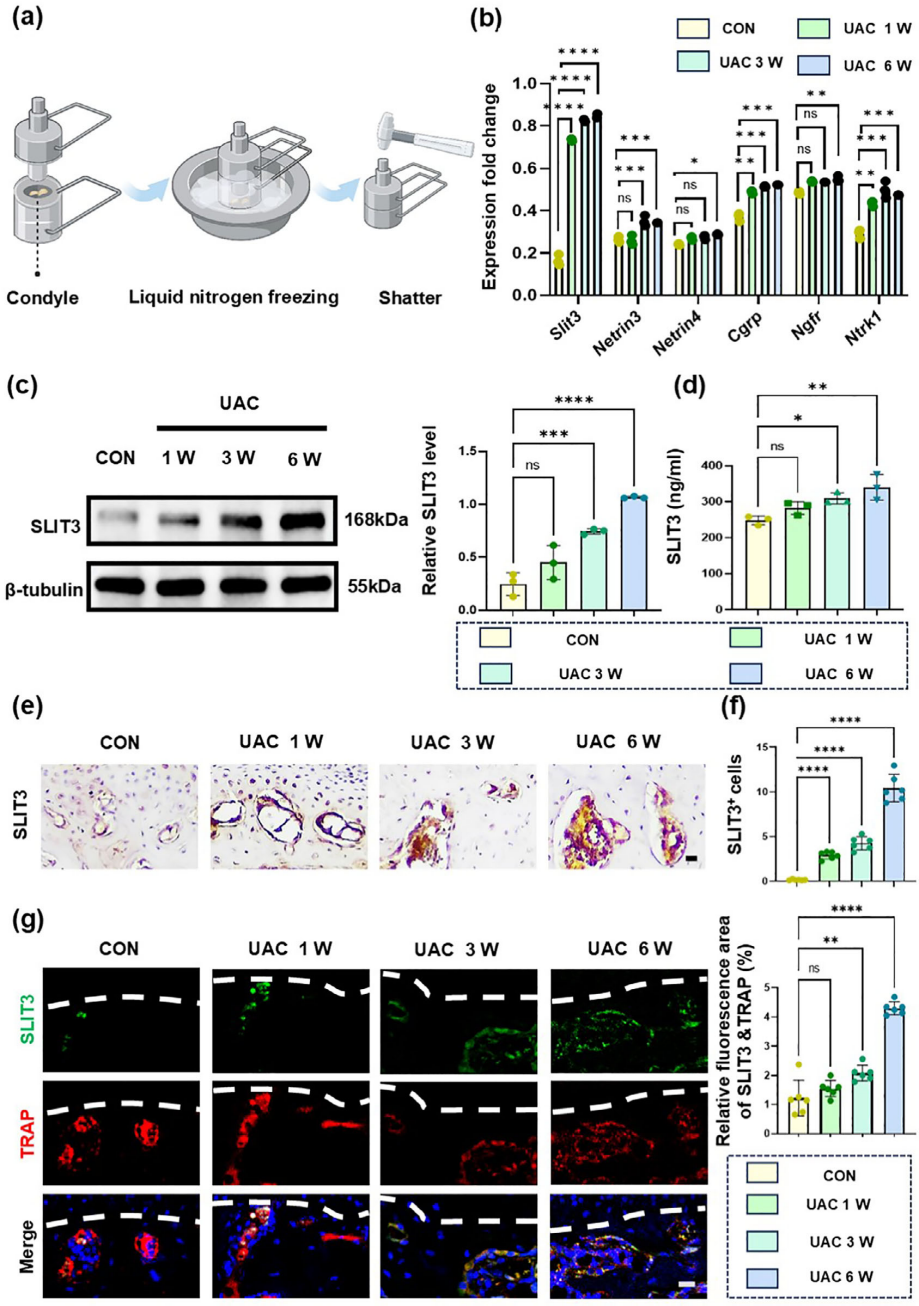
SLIT3 derived from osteoclasts mediates TMJ‐OA nerve growth. a) Schematic of experiment procedures. b) qRT‐PCR analysis of the gene expression of nerve growth factor (*Slit3, Ntn3, Ntn4, Cgrp, Ngfr, Ntrk1*) in the subchondral bone in four groups. *n* = 3. c) Western blot and d) ELISA results of SLIT3 expression in subchondral bone in CON and UAC groups. *n* = 3. e) Representative images of immunohistochemical staining of SLIT3 in subchondral bone. Scale bars: 70 µm. f) Quantitative analysis in panels (e) and (g). *n* = 6. g) Representative images of SLIT3 (green) and TRAP (red) co‐stained cells in subchondral bone in the CON and UAC groups. The white dashed line represents the boundary between bone and cartilage. *n* = 6. Scale bars: 10 µm. Statistical analyses were performed using one‐way ANOVA with Holm–Šidák multiple comparison tests. ^*^ *p* < 0.05. ^**^ *p* < 0.01. ^***^ *p* < 0.001. ^****^ *p* < 0.0001. *ns*: no significance.

In the mammalian nervous system, the connection of neuronal axons to tissues is guided by specific signals in the extracellular environment, a process known as axon guidance.^[^ ^37^ ^]^ To investigate the mechanism by which osteoclasts induced axon growth, we conducted UAC modeling on Six‐week‐old female Sprague–Dawley (SD) rats (weighing 140 to 160 g) and subsequently collected samples of the condyle at different times. After peeling off chondrocytes under a stereomicroscope, we tested the expression of a series of neurotrophic factors in the subchondral bone of the different groups. The results showed that the expression of *Slit3* gradually increased with prolonged modeling time (**Figure** **4**a,b; Table S1, Supporting Information). This observation was subsequently confirmed by western blotting analysis (Figure 4c), ELISA (Figure 4d), and immunohistochemical staining (Figure 4e,f). As a recognized neurotrophic factor, SLIT3 regulates nerve growth under various physiological conditions. Moreover, the growth, development, and repair of bones depend on proper innervation, and the physical and functional connections between the nervous system and the skeletal system form the neuroskeletal network.^[^ ^38^ ^]^ Osteoclasts and osteoblasts are key cells regulating the progression of TMJ‐OA.^[^ ^39^ ^]^

**Figure 5:**
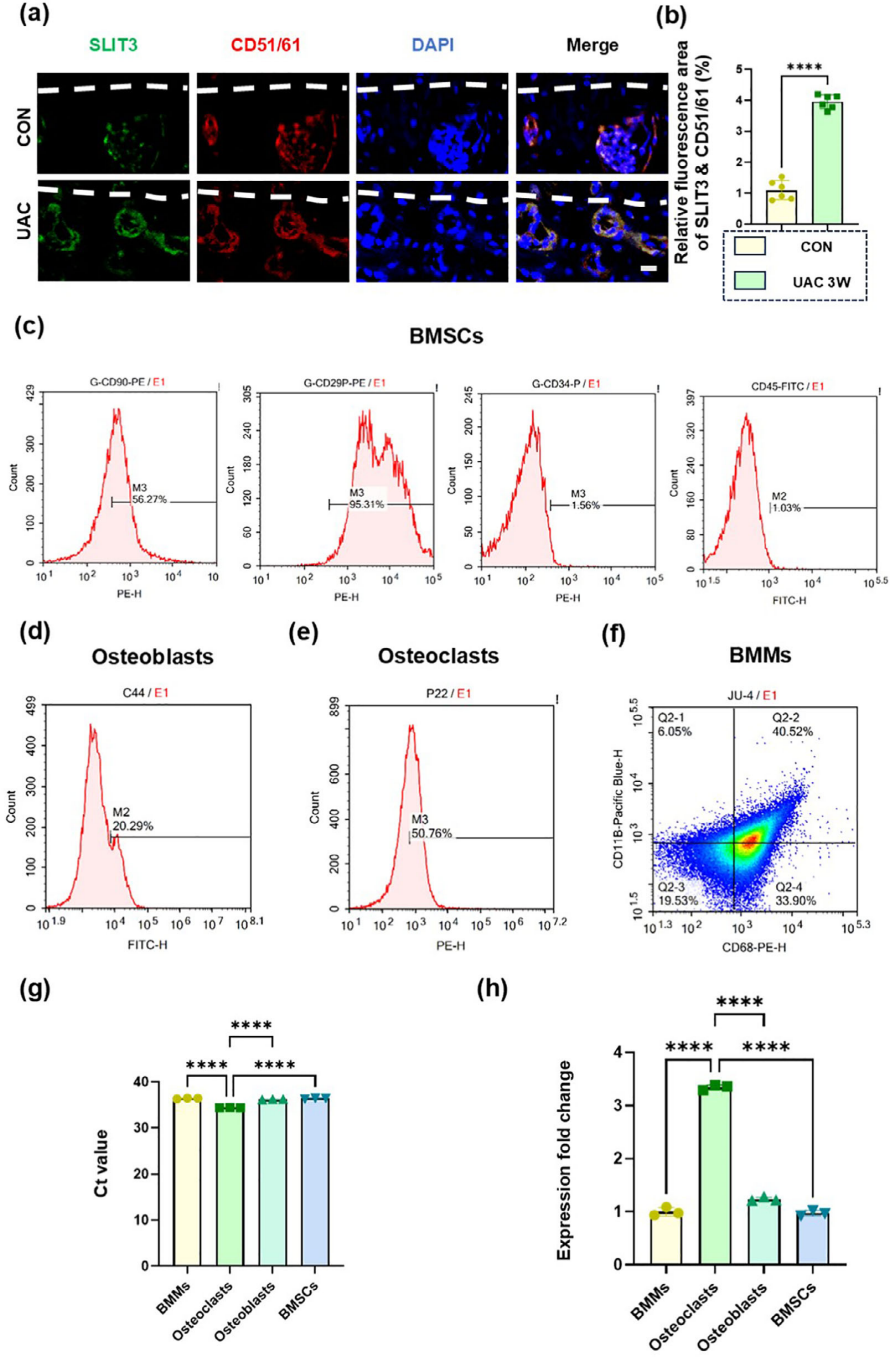
SLIT3 is mainly derived from osteoclasts. a) Representative images of SLIT3 (green) and CD51/61 (red) co‐stained cells in subchondral bone in the CON and UAC groups. The white dashed line represents the boundary between bone and cartilage. Scale bars: 10 µm. b) Quantitative analysis in panel (a). *n* = 6. c–e) FACS of the markers of BMSCs (c), osteoblasts (d), osteoclasts (e) and BMMs. f) from the mandible in 6‐week‐old female UAC rats. *n* = 6. g) The average Ct value and h) qRT‐PCR analysis of the expression of the *Slit3* in BMSCs, osteoblasts, osteoclasts, and BMMs. *n* = 3. Statistical analyses were performed using Student's *t*‐test. ^****^ *p* < 0.0001. *ns*: no significance.

To confirm the source of SLIT3, we performed co‐localization staining of SLIT3 with TRAP or osteocalcin on the same tissue sections, and the percentage of double‐positive cells relative to the total area was calculated for intergroup comparisons. The results showed that both osteoclasts and osteoblasts produced SLIT3 in the early stages of disease progression; however, osteoclasts expressed higher levels of SLIT3 (Figure 4g; Figure S3, Supporting Information). SLIT3 controls axonal guidance and neuronal migration by binding to Drosophila roundabout homologs (ROBOs) on the cell membrane.^[^ ^40^ ^]^ It has been previously reported that SLIT3 acted via the ROBO1 receptor to activate the Protein Kinase A (PKA) pathway and stimulate neural growth.^[^ ^41^ ^]^ In our study, double immunofluorescence staining of the TMJ subchondral bone for SLIT3 and ROBO1 demonstrated their co‐localization and a significant, time‐dependent upregulation in the UAC group compared to the controls (Figure S4, Supporting Information). Furthermore, considering that SLIT3 and TRAP are all secretory proteins, we further stained the membrane specific marker CD51/61 of osteoclasts, with SLIT3 for additional immunofluorescence staining, to accurately reflect the cellular origin of SLIT3 in CON and UAC groups (**Figure** **5**a,b).^[^ ^42^ ^]^ The result demonstrated substantial SLIT3 expression in CD51/61‐positive osteoclasts, consistent with the co‐localization of TRAP and SLIT3. Taken together, these results further confirmed that SLIT3 was indeed expressed by osteoclasts. In addition, to precisely identify the cellular origin of SLIT3, we conducted fluorescence‐activated cell sorting (FACS) on the mandibles of rats three weeks after modeling to isolate and analyze distinct bone marrow cell populations, including bone marrow‐derived macrophages (BMMs), osteoclasts, bone marrow stromal cells (BMSCs) and osteoblasts (Figure 5c–f). The results of FACS, together with subsequent quantitative real‐time polymerase chain reaction (qRT‐PCR) analysis, demonstrated that *Slit3* expression was predominantly detected in osteoclasts, with little expression in other cell lineages (Figure 5g,h; Table S2, Supporting Information). The amplification products demonstrated specificity, as confirmed by a single peak in the RT‐qPCR dissociation curve (Figure S5, Supporting Information) and a distinct band of the expected size (145 bp) on agarose gel electrophoresis (Figure S6, Supporting Information). Therefore, we hypothesized that in TMJ‐OA, osteoclast‐derived SLIT3 played a significant role in regulating nerve growth and pain.

**Figure 6:**
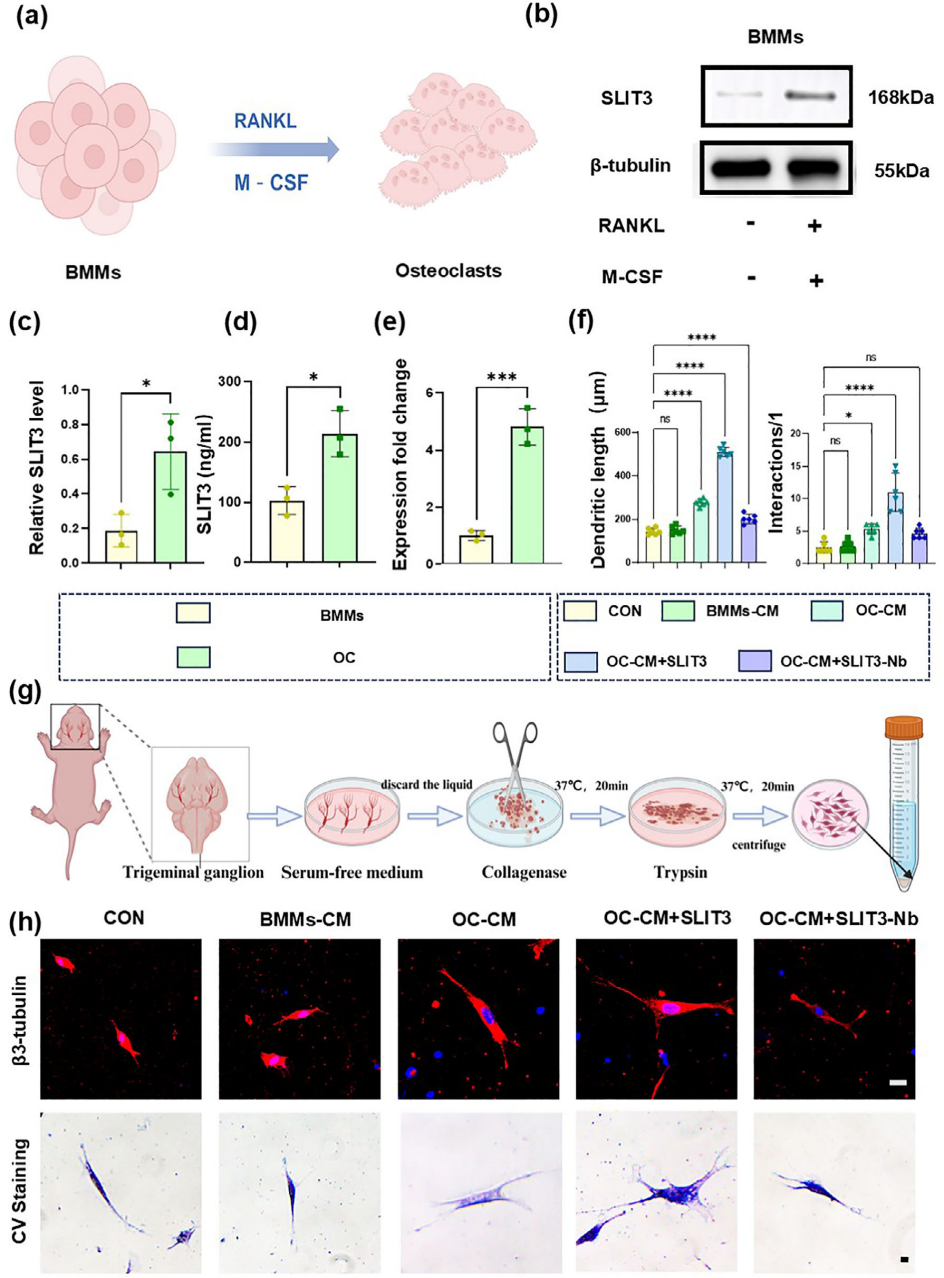
Confirmation of the effect of SLIT3 on sensory nerves through in vitro experiments. a) Schematic diagram of osteoclasts induction. b) Western blot of SLIT3 expression in bone BMMs and mature osteoclasts. c) Quantitative analysis of Western blot in panel (b). *n* = 3. d,e) ELISA (d) and qRT‐PCR analysis (e) in BMMs and mature osteoclasts. *n* = 3. f) Quantitative analysis in panel (h). *n* = 6. g) Schematic diagram for extracting trigeminal ganglion (TG) cells. h) Representative microscopy images of TG cells after different treatments. Scale bars: 10 µm. Statistical analyses were performed using Student's *t*‐test in (c,d,e) and one‐way ANOVA with Holm–Šidák multiple comparison tests in (f). ^*^ *p* < 0.05. ^***^ *p* < 0.001. ^****^ *p* < 0.0001. *ns*: no significance.

To further verify the regulatory effect of osteoclast‐derived SLIT3 on sensory nerves, we established an in vitro model of trigeminal ganglion (TG) cells. Above all, we induced the formation of mature osteoclasts (**Figure** **6**a; Figure S7, Supporting Information) and confirmed the significant expression of SLIT3 in these osteoclasts using Western Blotting analysis (Figure 6b,c), ELISA (Figure 6d), and qRT‐PCR (Figure 6e). Crystal violet staining and immunofluorescence results showed that the axons of sensory neurons cultured in the supernatant of BMMs did not change significantly compared with those cultured alone, while the axons of sensory neurons co‐cultured with the supernatant of osteoclasts exhibited significantly enhanced elongation and diffusion (Figure 6f–h). These findings confirmed the regulatory effect of osteoclasts on sensory nerves. To further verify the role of osteoclasts, we added 500 µg mL^−1^ SLIT3 protein to the co‐culture medium in vitro, which significantly promoted the growth of sensory neuron axons (Figure 6f–h). To further verify the role of SLIT3, we used 5 µg mL^−1^ neutralizing antibodies against SLIT3, which inhibited the growth advantage of sensory nerve axons in the co‐culture system (Figure 6f–h). The above results indicated that osteoclast‐derived SLIT3 had a regulatory effect on the growth of sensory nerves. Overall, the above results suggest that osteoclast‐derived SLIT3 can significantly promote the growth of sensory nerve axons.

### 2.4 TRAP^+^ Osteoclast‐Derived SLIT3 Mediates TMJ‐OA Pain

**Figure 7:**
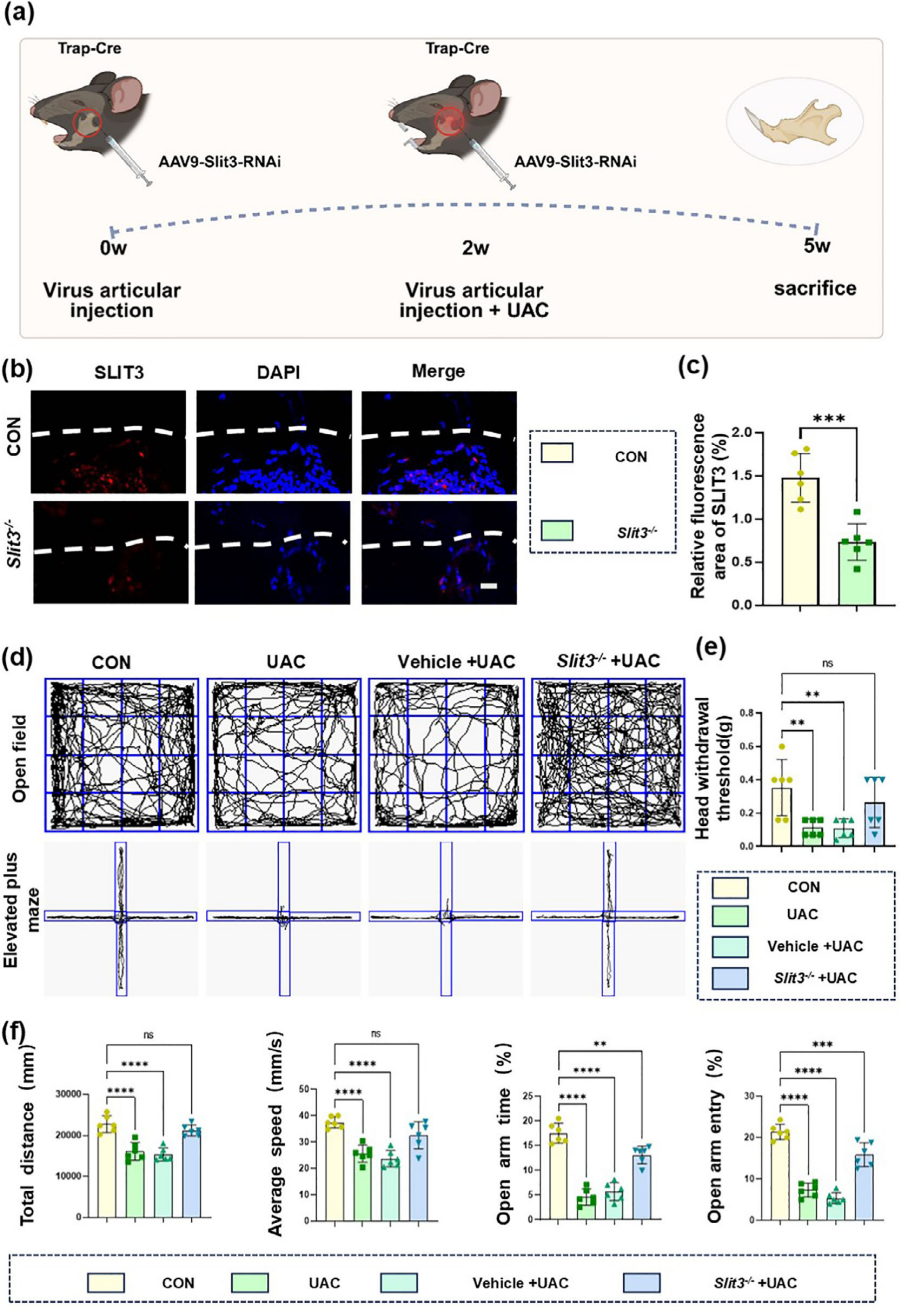
Pain in TRAP‐Cre; *Slit3^−/−^* mice is reduced. a) Experimental design for TRAP‐Cre transgenic mice injected with AAV9‐Slit3‐RNAi. b) Immunofluorescence of SLIT3 (red) in condyles. The white dashed line represents the boundary between subchondral bone and cartilage. Scale bars:10 µm. c) Quantitative analysis in panel (b). *n* = 6. d) Representative results of OFT and EPM. e,f) Quantitative analysis of Von Frey experiment (e), OFT and EPM (f). *n* = 6. Statistical analyses were performed using Student's *t*‐test in (c), and one‐way ANOVA with Holm–Šidák multiple comparison tests in (e,f). ^**^ *p* < 0.01. ^***^ *p* < 0.001. ^****^ *p* < 0.0001. *ns*: no significance.

We found that during the development of TMJ‐OA, the activity of TRAP^+^ osteoclasts increased significantly, promoting the ingrowth of sensory nerves at the osteochondral junction via the secretion of SLIT3. To further verify the role of TRAP^+^ osteoclast‐derived SLIT3 in vivo, we used TRAP‐Cre transgenic mice combined with AAV9‐*Slit3*‐RNAi^[^ ^43^, ^44^, ^45^ ^]^ (Figure S8, Supporting Information) to construct *Slit3*‐targeted knockdown mice and performed TMJ‐OA modeling (**Figure** **7**a). The immunofluorescence results demonstrated that the SLIT3 level of the targeted knockdown group was significantly lower than that in the control group (Figure 7b,c). This demonstrated that the TRAP‐Cre; *Slit3* ^−/−^ mice were successfully constructed. To verify the effect of TRAP^+^ osteoclast‐derived SLIT3 on pain, we conducted Von Frey tests, OFT and EPM on four groups of mice. The results showed that knocking down *Slit3* in TRAP^+^ osteoclasts significantly reduced the pain behavior of OA mice (Figure 7d–f). The above results indicated that SLIT3 derived from TRAP^+^ osteoclasts is associated with the occurrence of pain during the progression of TMJ‐OA.

**Figure 8:**
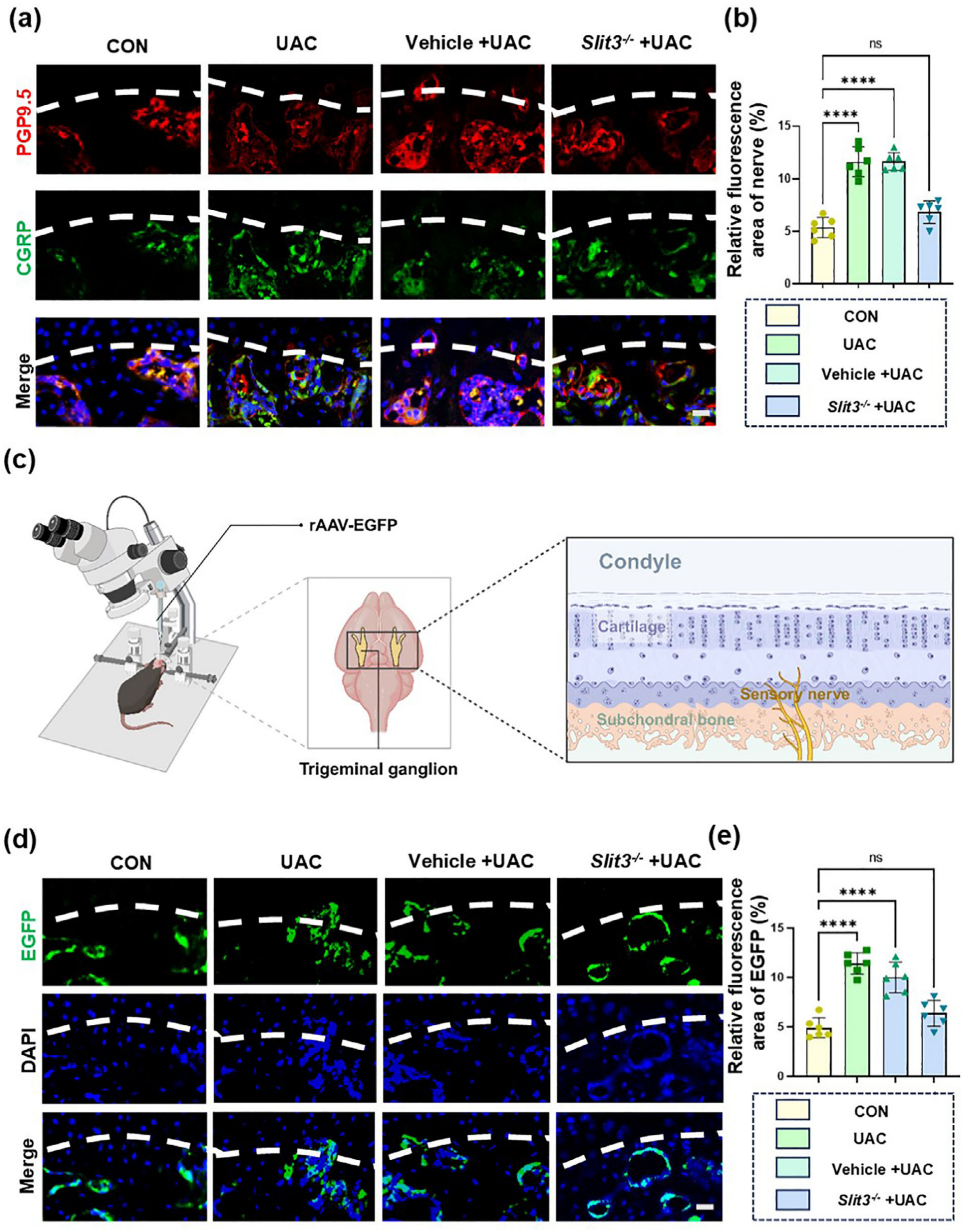
Immunofluorescence imaging and analysis of sensory nerve distribution in the condyle of *Slit3*‐knockdown mice. a) Representative images of immunofluorescence staining for PGP9.5 (red), CGRP (green) and DAPI (blue) along the TMJ osteochondral junction. Scale bars: 10 µm. b) Quantitative analysis of the density of PGP9.5^+^ nerve fibers and CGRP^+^ nerve fibers in the TMJ osteochondral junction. *n* = 6. c) Schematic diagram of anterograde tracing. d) Representative images of the condylar sensory nerve. EGFP (rAAV‐CAG‐EGFP‐WPRE‐hGH pA, green), DAPI (blue). Scale bars: 10 µm. e) Quantitative analysis of the density of sensory nerve fibers. *n* = 6. The white dashed line represents the boundary between bone and cartilage. Statistical analyses were performed using one‐way ANOVA with Holm–Šidák multiple comparison tests. ^****^ *p* < 0.0001. *ns*: no significance.

Immunofluorescence staining of PGP9.5 and CGRP was performed to study the distribution of sensory nerves at the osteochondral junction. The results demonstrated that in the UAC group, the sensory nerves in the subchondral bone penetrated the noncalcified cartilage layer, disrupting the integrity of the osteochondral connection (**Figure** **8**a,b). After knocking down *Slit3*, this pathological phenomenon was inhibited. This suggested that SLIT3 derived from TRAP^+^ osteoclasts could induce the innervation of sensory nerves. Anterograde tracing experiments using rAAV‐CAG‐EGFP‐WPRE‐hGH pA injection into TG further verified these results (Figure 8c–e).

### 2.5 TRAP^+^ Osteoclast‐Derived SLIT3 Aggravates Cartilage Degradation and Increases Subchondral Bone Loss in TMJ‐OA

**Figure 9:**
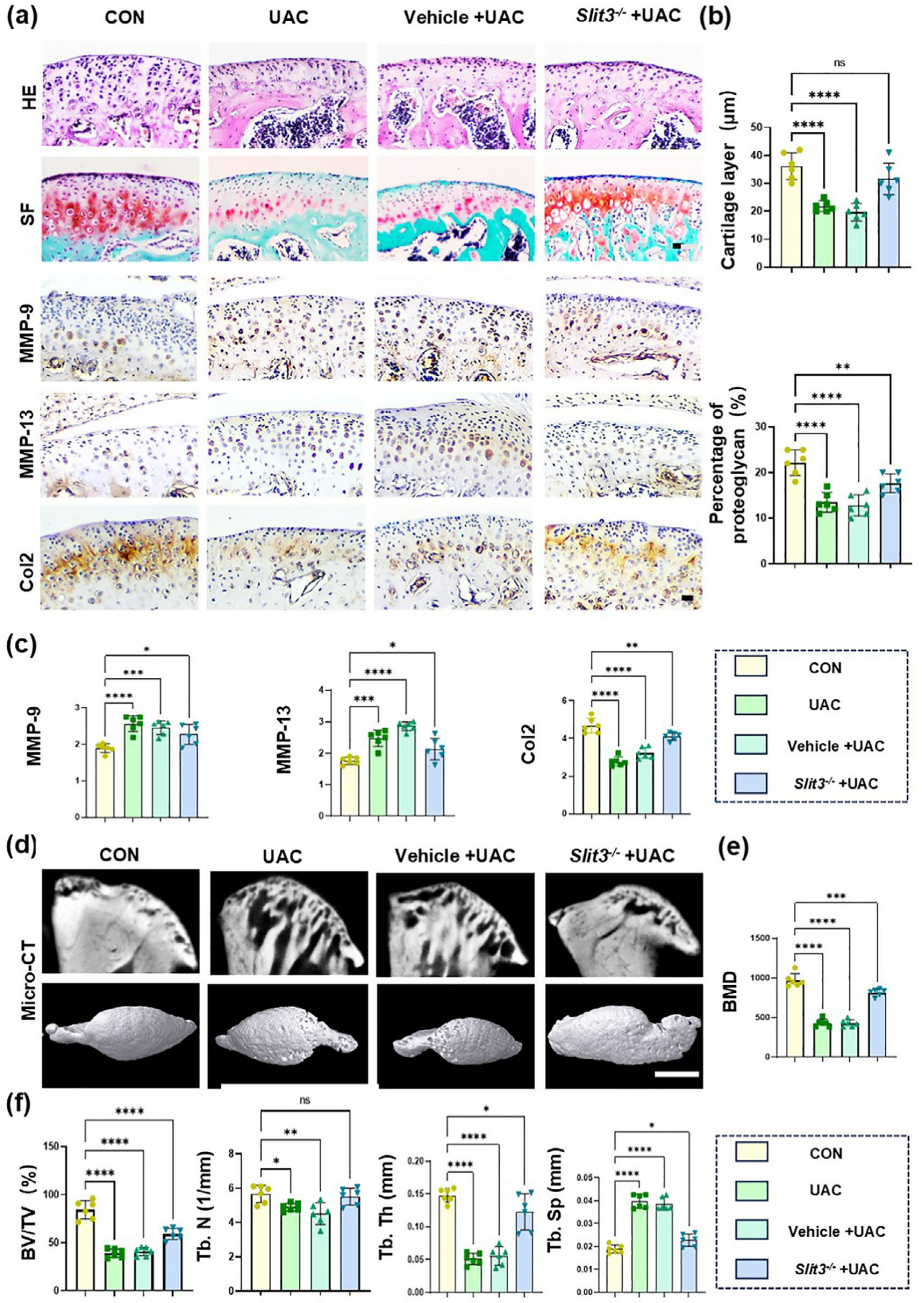
Histological staining and analysis of the condyle of *Slit3*‐knockdown mice. a) Representative images of HE, SF and immunohistochemical staining (MMP‐9, MMP‐13, Col2) of the mandibular condyle. Scale bars:70 µm. b,c) Quantitative analysis of HE staining, SF staining and immunohistochemical staining. *n* = 6. d) Micro‐CT scan of the condylar subchondral bone. Scale bars:200 µm. e,f) Quantitative analysis of micro‐CT data on BMD, BV/TV, Tb.Th, Tb.N and Tb.Sp. *n* = 6. Statistical analyses were performed using one‐way ANOVA with Holm–Šidák multiple comparison tests. ^*^ *p* < 0.05. ^**^ *p* < 0.01. ^***^ *p* < 0.001. ^****^ *p*<0.0001. *ns*: no significance.

The ingrowth of nerves into noncalcified cartilage disrupts the integrity of the connection between articular cartilage and subchondral bone.^[^ ^46^ ^]^ Histological staining showed that in the UAC group and Vehicle + UAC group, the density and thickness of cartilage and proteoglycans significantly decreased, and cell‐free areas appeared in the condylar cartilage (**Figure** **9**a,b). In the *Slit3* ^−/−^ group, the cartilage thickness and proteoglycan area increased significantly (Figure 9a,b). Further detection showed that the UAC group exhibited much more factors related to cartilage. Next, we performed immunohistochemical staining, in the proliferative and hypertrophic layers of the condyle in control group mice, fewer cells positive for MMP‐9 and MMP‐13 were observed in the cartilage, and Type‐II collagen (Col2) was evenly distributed. In the UAC group and Vehicle + UAC group, the number of MMP‐9 and MMP‐13 positive cells increased significantly, while the percentage area of Col2 decreased significantly. In the *Slit3* ^−/−^ group, cartilage degradation was significantly inhibited (Figure 9a,c). Overall, knocking down *Slit3* in TRAP^+^ osteoclasts significantly slowed the process of cartilage degradation in TMJ‐OA.

In TMJ‐OA, when cartilage degradation occurs, subchondral bone remodeling leads to bone loss. Micro‐CT analysis showed that in UAC groups, the BV/TV, BMD, Tb.N, and Tb.Th decreased significantly, while the Tb.Sp doubled compared with that in the control group. In the *Slit3* ^−/−^ group, these parameters significantly increased compared with those in the UAC group, while the Tb.Sp decreased (Figure 9g–i). The above results indicated that in the progression of TMJ‐OA, SLIT3 derived from TRAP^+^ osteoclasts increased bone loss in the subchondral bone, while knocking down *Slit3* improved this symptom.

### 2.6 Conditional Deletion of *Slit3* in Osteoclast Attenuated TMJ‐OA Pain and Nerve growth

**Figure 10:**
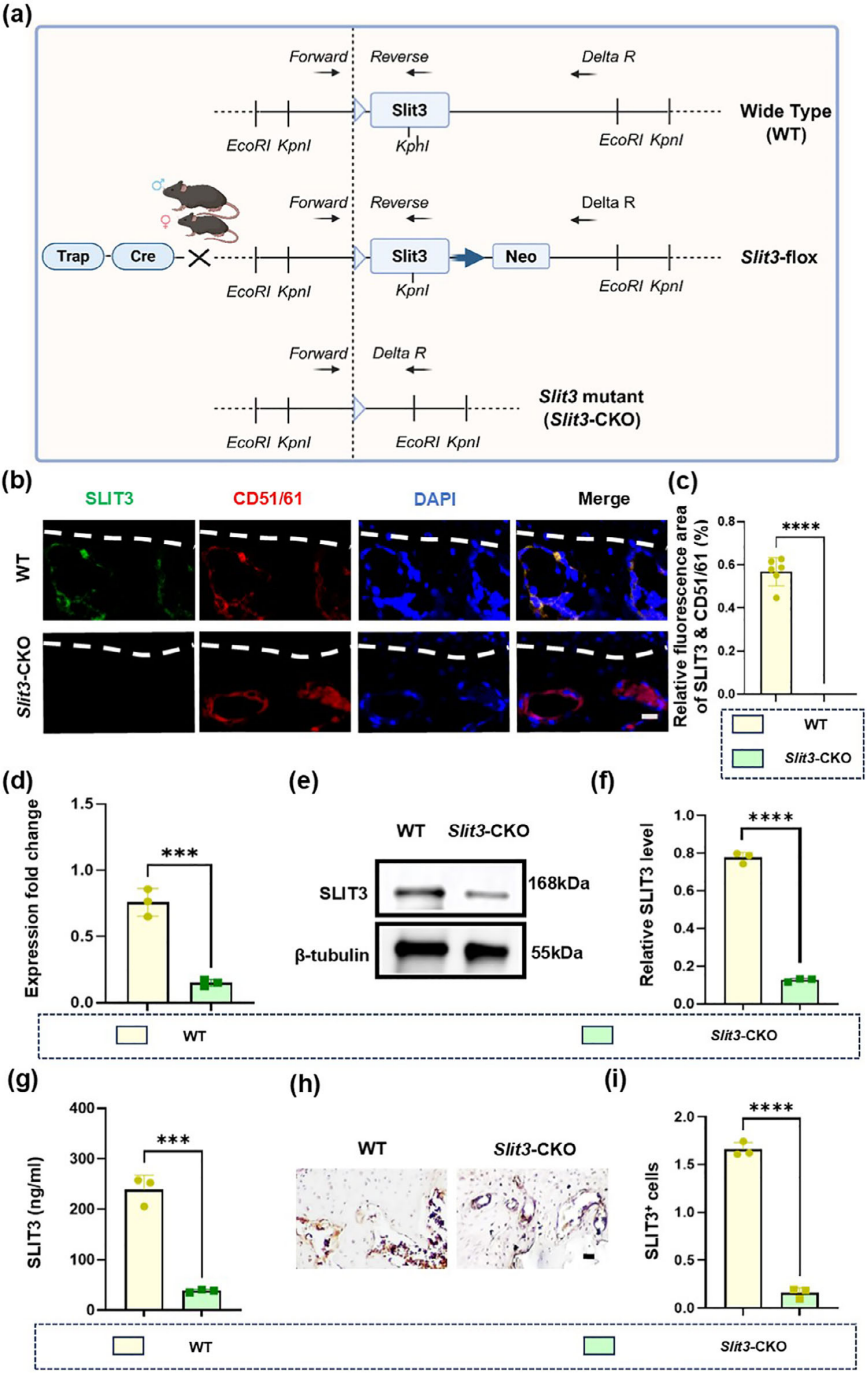
*Slit3* is specifically knocked out in osteoclasts of *Slit3*‐CKO mice. a) Schematic diagram of *Slit3*‐CKO mice. b) Immunofluorescence of SLIT3 (green) and CD51/61 (red) in subchondral bone. The white dashed line represents the boundary between subchondral bone and cartilage. Scale bars:10 µm. c) Quantitative analysis in panel (b). *n* = 6. d) qRT‐PCR analysis of the gene expression of *Slit3* in subchondral bone in the two groups. *n* = 3. e,f) Western blot and its quantitative analysis, g) ELISA, and h,i) immunohistochemical staining and quantitative analysis of SLIT3 expression in subchondral bone in the WT and *Slit3*‐CKO groups. *n* = 3. Scale bars: 70 µm. Statistical analyses were performed using Student's *t*‐test. ^*^ *p* < 0.05. ^**^ *p* < 0.01. ^***^ *p* < 0.001. ^****^ *p* <0.0001. *ns*: no significance.

Our findings revealed that specific knockdown of *Slit3* in the osteoclasts resulted in reduced sensory nerves within the condylar cartilage, leading to a significant decrease in pain. Additionally, both bone and cartilage loss were mitigated by knockdown of *Slit*3. Next, to further determine the role of SLIT3 in osteoclasts, we crossed TRAP‐Cre mice with *Slit3* ^flox/flox^ mice (Figure S9, Supporting Information) to produce the osteoclast specific *Slit3* knockout mice (**Figure** **10**a). To confirm the expression of SLIT3, we performed co‐localization staining of SLIT3 with CD51/61. The results showed that SLIT3 derived from osteoclasts was knocked out (Figure 10b,c). We detected the expression of *Slit3* and the results showed that its expression was significantly reduced in *Slit3*‐CKO mice (Figure 10d). Besides, western blot analysis (Figure 10e,f), ELISA (Figure 10g), and immunohistochemical staining (Figure 10h,i) also confirmed a decrease in SLIT3 levels.

**Figure 11:**
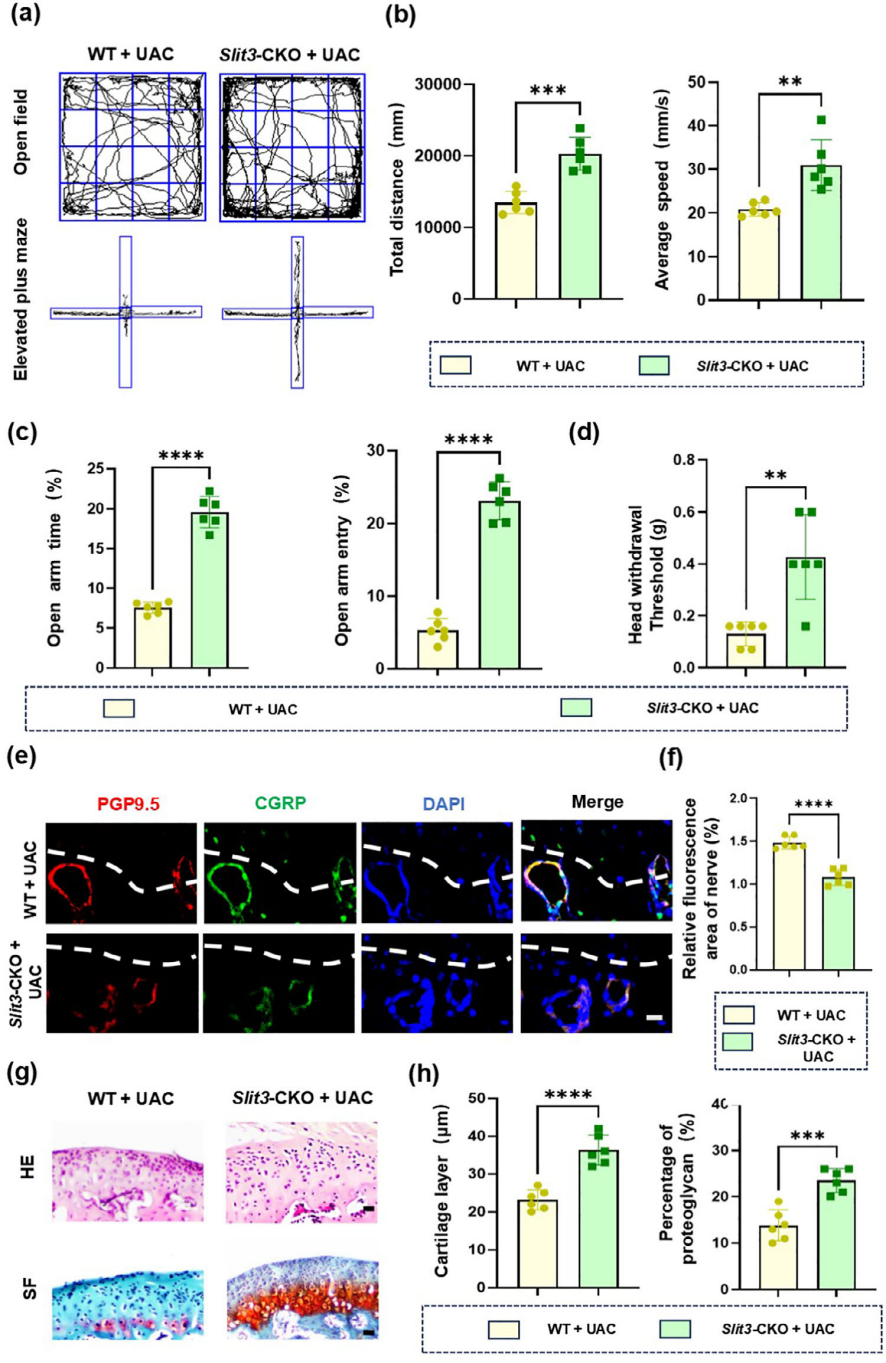
Conditional deletion of *Slit3* in Osteoclast attenuated TMJ‐OA Pain and Nerve growth. a) Representative results of OFT and EPM. b,c) Quantitative analysis in panel (a). *n* = 6. d) Quantitative analysis of Von Frey experiment. *n* = 6. e) Representative images of PGP9.5 (red) and CGRP (green) co‐stained cells along the TMJ osteochondral junction in the WT + UAC and *Slit3*‐CKO + UAC groups. Scale bars: 10 µm. f) Quantitative analysis in panel (e). *n* = 6. g) Representative images of HE and SF staining of the mandibular condyles. Scale bars:70 µm. h) Quantitative analysis in panel (g). *n* = 6. Statistical analyses were performed using Student's *t*‐test. ^**^ *p* < 0.01. ^***^ *p* < 0.001. ^****^ *p* < 0.0001.

To examine the effects of osteoclast‐specific *Slit3* conditional deletion on pain and nerve growth, we developed UAC models in both wide type (WT) and *Slit3*‐CKO groups. The Von Frey tests, OFT and EPM were performed in both groups. Our findings demonstrated that pain‐related behaviors were markedly attenuated in *Slit3*‐CKO mice (**Figure** **11**a–d). The immunofluorescence staining of PGP9.5 and CGRP showed that in the WT + UAC group, sensory nerves in the subchondral bone penetrated into noncalcified cartilage layer, disrupting the integrity of the bone cartilage connection (Figure 11e,f). After knocking out *Slit3*, the growth of sensory nerves at the TMJ osteochondral junction was inhibited. This indicated that knocking out SLIT3 in TRAP^+^ osteoclasts significantly reduced the growth of sensory nerves (Figure 11e,f). Histological staining revealed that knocking out *Slit3* in osteoclasts significantly slowed the process of cartilage degradation in TMJ‐OA (Figure 11g,h). These results collectively suggest that SLIT3 secreted by TRAP^+^ osteoclasts contributes to pain pathogenesis during TMJ‐OA progression.

## 3 Conclusion

The cellular origin of SLIT3 in long bones remains unclear, although some studies suggest that osteoclasts are the primary source of SLIT3.^[^ ^26^, ^47^ ^]^ In our study, we found that increased osteoclast activation and SLIT3 expression in the subchondral bone were accompanied with OA pain, sensory nerve innervation and joint degeneration. To further verify that osteoclasts are the main cause of SLIT3 in the mandible, a kind of flat bone, we conducted FACS to isolate and analyze distinct bone marrow cell populations, including BMMs, osteoclasts, BMSCs and osteoblasts. Subsequent qRT‐PCR analysis with detailed cycle threshold values demonstrated that SLIT3 expression was predominantly detected in osteoclasts. Finally, we specifically intervened *Slit3* (encoding slit guidance ligand 3) in TRAP^+^ osteoclasts by an AAV knockdown technology in TRAP‐Cre transgenic mice and TRAP‐Cre mice with *Slit3* ^flox/flox^ mice (C57BL/6Smoc‐Slit3^em1(flox)Smoc^), and the results demonstrated that the specific interventions of *Slit3* in osteoclasts significantly reduced the level of SLIT3 in subchondral bone. Developed from neuroectoderm, TMJ exhibits significant peripheral nervous regulation in development.^[^ ^48^, ^49^ ^]^ Therefore, our findings validated that the osteoclasts from the subchondral bone of TMJ condyle indeed expressed SLIT3, and highlighted the critical role of SLIT3 from TRAP^+^ osteoclasts in the progression of TMJ‐OA and the development of associated pain.

Abnormal subchondral bone remodeling and the structural deterioration within the subchondral bone play crucial roles in the development and progression of TMJ‐OA.^[^ ^31^ ^]^ The regulation of bone homeostasis cannot be separated from the monitoring and regulation of the nervous system. For example, previous studies found that the inflammatory response caused by peripheral bone trauma was transmitted to the central nervous system through peripheral sensory nerves, and further affected bone tissue repair through sympathetic nervous system regulation.^[^ ^50^, ^51^ ^]^ Under conditions of bone homeostasis disruption, inflammatory cells infiltrated osseous and osteochondral tissues, releasing proinflammatory cytokines that contributed to pain‐related phenotypes. Concurrently, aberrant osteoclast activation promoted excessive secretion of Netrin‐1 by osteoclast precursors, inducing nociceptive sensory nerve ingrowth into the vertebral endplate and subchondral bone, thereby driving low back pain and joint pain.^[^ ^52^ ^]^ Furthermore, bisphosphonates played a role in regulating the nervous system by inhibiting osteoclast activation, promoting osteogenic regulation and bone remodeling.^[^ ^53^ ^]^

Studies have shown that SLIT3/ROBO2 modulated endochondral ossification in bone development, and that it mediated hypertrophic chondrocyte maturation via suppression of β‐catenin activity. Our results also confirmed that chondrocyte hypertrophy was markedly attenuated in *Slit3* ^−/−^ mice. The knockdown of *Robo2* with its siRNA completely reversed the SLIT3‐stimulated MMP‐13 expression in chondrocyte.^[^ ^54^ ^]^ Furthermore, *Slit3* silencing restrained articular cartilage degradation, aberrant subchondral bone formation, and H‐type vessel formation in OA mice. Inhibition5 of Slit3/Robo1 signaling alleviated osteoarthritis in mice by reducing abnormal H‐type vessel formation in the subchondral bone.^[^ ^55^ ^]^ Our findings demonstrate that targeted intervene of *Slit3* in TRAP^+^ osteoclasts significantly ameliorates pathological subchondral bone remodeling and cartilage degeneration. The observed therapeutic effect may be attributed to the reversal of abnormal bone remodeling after *Slit3* knockout, which improves the biomechanical microenvironment of articular cartilage, thereby promoting cartilage repair and protection. In addition, the decreased SLIT3 concentration at the osteochondral junction in the OA joints might alleviate its destructive effects on the cartilage as previous reported.^[^ ^54^, ^55^ ^]^ Taken together, our study provides compelling evidence that TRAP^+^ osteoclasts serve as the predominant source of SLIT3 in TMJ‐OA pathogenesis, and that osteoclast‐derived SLIT3 plays a pivotal role in driving the progression of osteochondral lesions in OA joints. In future studies, we plan to employ single‐cell RNA sequencing to further validate the expression of SLIT3 in osteoclasts.

In the growth and development of nerves, SLIT3 activates one of the most important repulsive axonal guidance signaling pathways in nervous system development by interacting with axon guidance receptors (Drosophila roundabout homologs, ROBOs) on the cell membrane, to control axonal pathfinding and neuronal migration.^[^ ^40^ ^]^ A previous study showed that SLIT3 acted on the ROBO1 receptor on nerves, activating the protein kinase A (PKA) signaling pathway and promoting nerve growth by phosphorylating tyrosine hydroxylase through the PKA/calcium/calmodulin dependent protein kinase (CaMKII) pathway.^[^ ^41^ ^]^ The ROBO family of receptors, including ROBO1–4, exhibits different expression patterns in various nerve cells and participates in the process of nerve growth.^[^ ^40^, ^56^, ^57^ ^]^ Our results demonstrate a significant positive correlation between elevated subchondral bone SLIT3 levels and neuronal growth/cartilage degeneration during TMJ‐OA progression. Notably, knockout of SLIT3 from TRAP^+^ osteoclast resulted in significant reversal of sensory nerve innervation and pain‐related behavior in TMJ‐OA mice, strongly suggesting that osteoclast‐secreted SLIT3 plays a pivotal role in regulating axonal outgrowth within the subchondral bone microenvironment. The observed cartilage‐protective effects likely occur through SLIT3‐mediated modulation of subchondral bone remodeling, which promotes cartilage phenotype restoration by improving the biomechanical microenvironment. In addition, the decreased SLIT3 concentration in the osteochondral regions in OA joints might alleviate the destructive effects of SLIT3 on cartilage as previous reported.^[^ ^54^, ^55^ ^]^ In the future, we will investigate the specific mechanisms by which SLIT3 regulates the nerve growth and cartilage degeneration in the progress of TMJ‐OA.

The production of SLIT3 is regulated by the mitotic deacetylase complex (MiDAC). During neural differentiation and axonal elongation in mouse embryonic stem cells, MiDAC deacetylates histone 4 lysine 20 (H4K20), thereby activating the promoter and enhancer regions of the *Slit3* and *Ntn1* genes. Meanwhile, it inhibits the transcription of genes encoding negative regulatory factors such as sprouty RTK signaling antagonist 4 (Spry4) and inhibitor of DNA binding 1 (Id1) in neurogenesis.^[^ ^58^ ^]^ The Wnt signaling pathway plays a crucial role in the axonal pathways of the forebrain, and research on early neural development in zebrafish has shown that the expression of SLIT3 was inhibited by the Wnt signaling pathway.^[^ ^59^ ^]^ In addition to using relevant inhibitors, modulating the signals generated by MiDAC or enhancing the inhibitory Wnt signal can help reduce SLIT3 production.^[^ ^58^, ^59^ ^]^ These findings provide potential targets for therapeutic interventions aimed at reducing SLIT3 levels and mitigating the associated pain and nerve growth in TMJ‐OA.

In this study, our group innovatively constructed an animal model of TMJ‐OA pain, laying a solid foundation for future research on the mechanisms of joint pain.^[^ ^29^ ^]^ We first examined the histopathological changes in the condyles of mice using HE and SF staining. Subsequently, the von Frey test results demonstrated that the TMJ‐OA mice had a lower pain threshold and displayed significantly more pain behavior compared with the control group. Additionally, a previous study showed that mice with chronic pain not only exhibited pain‐related behaviors but also showed signs of depression.^[^ ^28^ ^]^ Therefore, we also conducted EPM and OFT, which are related to depression. This comprehensive approach validated the successful establishment of the TMJ‐OA pain model, and provided insights into the behavioral and psychological aspects of chronic pain. Actually, the experimental approach utilizing viral‐mediated gene knockdown in Cre‐expressing mice has been widely employed.^[^ ^60^, ^61^, ^62^ ^]^ Our results obtained from TRAP‐Cre; Slit3^flox/flox^ conditional knockout mice are consistent with the AAV9‐Slit3‐RNAi methodology. Specifically, both approaches demonstrate that targeted deletion of *Slit3* in TRAP^+^ osteoclasts significantly reduces SLIT3 expression in subchondral bone and effectively reverses the observed OA pathological phenotype. This concordance between two independent experimental systems strengthens the validity of our conclusions regarding the crucial role of osteoclast‐derived SLIT3 in subchondral bone pain and nerve growth during TMJ‐OA progression. However, TRAP is not exclusively expressed by osteoclasts, we will use CTSK‐Cre; SLIT3^−/−^ mice to further validate this finding in future studies. To explore the clinical translational potential of targeting SLIT3, further studies are needed to use a SLIT3‐neutralizing antibody to further corroborate our genetic evidence and to directly test its therapeutic efficacy in alleviating TMJ‐OA pain.

In summary, this study provides a theoretical framework for the treatment of TMJ‐OA and other bone pain‐related diseases, suggesting that targeting SLIT3, osteoclasts, and related signaling pathways could offer a comprehensive approach to manage these conditions.

## 4 Experimental Section

### Establishment of Animal Models

Animals were sourced from the Experimental Animal Center of Airforce Medical University (AFMU, Xi'an, China). All animal procedures were adhered to the guidelines of the Animal Care Committee of the AFMU, and all experimental protocols were approved by the AFMU. All applicable institutional and/or national guidelines for the care and use of animals were followed (Approval number: 20 250 077). In this study, 6‐week‐old C57BL/6J female mice, 6‐week‐old TRAP‐Cre female mice, 6‐week‐old *Slit3*‐CKO female mice, and 6‐week‐old female SD rats were used. And unilateral anterior crossbite (UAC) osteoarthritis models in different strains of mice were induced. In this study, all experimental groups (including the control and all UAC groups at different time points) had a sample size of *n* = 6, where “n” represents six individual mice or rats. For each animal, the left condyle was harvested and used for all subsequent experiments. The observation is based on six independent samples per group, with three regions analyzed per sample. For the establishment of UAC, two‐stage anesthesia was induced in mice. Then, a 1.5 mm metal tube was bonded to the left maxillary incisor using zinc phosphate cement and a 5 mm metal tube, bent at a 135° angle at one end, was bonded to the left mandibular incisor. In the control group, mice underwent the same anesthesia procedures without placement of the metal tubes. For the rat UAC model, the rats were also divided into a CON group and UAC group: For the experimental setup, two metal tubes were fabricated: a 3‐mm tube constructed from a 25‐gauge milk needle was affixed to the left maxillary incisor, while an 8‐mm tube made from a 20‐gauge milk needle, featuring a 135‐degree bend at one end, was secured to the left mandibular incisor to serve as a guide plate.

### TRAP‐Cre Transgenic Mice

Genetically modified mice expressing Cre recombinase specifically under the regulation of the *Trap* promoter were generated. The established TRAP‐Cre transgenic mouse line facilitates the targeted deletion of genes specifically in TRAP^+^ osteoclasts. The TRAP promoter source derived from the Jackson laboratory; Cre recombinase efficiency is 85%. Genomic DNA was extracted from mouse tail samples utilizing a rapid DNA isolation kit (D0065S, Beyotime, Haimen, China). Gene amplification was performed by qRT‐PCR using the following primers: Cre‐R (TTGCACGTTCACCGGCATCAACG) and Cre‐F (TTGCCTGCATTACCGGTCGATGC).

### Adeno‐Associated Virus (AAV9‐Slit3‐RNAi) Infection

AAV9‐*Slit3*‐RNAi (an AAV designed to exert RNA interference (RNAi) on *Slit3* and its control virus (GENE, China) were constructed and injected into the TMJ cavity of TRAP‐Cre transgenic mice to knockdown *Slit3* in TRAP^+^ osteoclasts. The Cre‐loxP system requires the simultaneous presence of both Cre and floxed elements. A viral vector encoding the mouse *Slit3* (NM_01 1412) was utilized. The construct (GV718) features the following elements: CMV bGlobin‐FLEX‐EGFP‐MIR155(mSlit3)‐WPRE‐Hgh polyA. This vector contains FLEX elements (Figure S8, page 7 in Supporting Information). The FLEX system introduces two different pairs of loxP sites. After inverting the target gene, these loxP sites can be excised, preventing the inversion reaction from reversing. This ensures that once the gene is inverted and expressed, the loxP sites are removed.^[^ ^63^ ^]^ When the present virus is injected into CRE mice, it can achieve gene knockdown.^[^ ^64^, ^65^ ^]^

### Intra‐articular Injection

All mice underwent a two‐stage anesthesia protocol: initial light anesthesia was induced using a 2% isoflurane‐oxygen mixture, followed by deep anesthesia administration through 10 mg mL^−1^ pentobarbital injection (30 mg kg^−1^). A Hamilton syringe was used, and the needle was inserted into the area below the zygomatic arch, between the eyes and ears, until it reacheed the outer surface of the mandibular ramus. The tip of the needle was guided along the bone wall to the TMJ area. The AAV9‐*Slit3*‐RNAi (0.30 × 10^−13^ g mL^−1^, diluted with phosphate‐buffered saline (PBS; catalog number AAV9CON545, GENE, China) and control virus (CON545, 0.15 × 10^−13^ g mL^−1^) were injected into the TMJ cavity of 6‐week‐old TRAP‐Cre mice at a dose of 10 µL/mouse. Two weeks later, AAV9‐*Slit3*‐RNAi (0.60 × 10^−13^ g mL^−1^) and control virus (CON545, 0.30 × 10^−13^ g mL^−1^) were injected again at the same dose (*n* = 6), followed by UAC model construction. In the control group, mice were injected with equivalent volumes of PBS. The RNAi target sequence was TTGTTTGCGACTGCCACTTGA. Samples were collected 3 weeks after modeling.

### Tissue Preparation and Histological Staining

Mice were euthanized by pentobarbital overdose. The TMJ condyles were separated from the mandible and fixed in 4% paraformaldehyde for 48 h. Decalcification was performed using 10% ethylenediaminetetraacetic acid (EDTA; pH 7.3) for 20 days, with EDTA replaced every 2 days. The samples were then embedded in paraffin and sectioned. It is worth noting that central sagittal sections of the temporomandibular joint (TMJ) were subjected to staining. Following this, the condylar cartilage image was divided into three equal‐width regions (anterior, middle, and posterior). A region of interest (ROI) was selected at the osteochondral junction within each region. The values from the three ROIs per sample were averaged to generate a single data point for statistical analysis (Figure S10, Supporting Information).

The sections were then stained with Hematoxylin–Eosin (HE) and safranin O‐fast green (SF). Following completion of staining, the sections were sequentially dehydrated through a graded ethanol series consisting of 70%, 80%, 95% (twice), and 100% (twice) ethanol, with 5‐min immersions at each concentration. Subsequently, the sections were cleared by immersion in xylene I and xylene II for 15 min each. Finally, the prepared sections were mounted using neutral balsam for microscopic examination. The sections were observed under an optical microscope (Leica Microsystems, Wetzlar, Germany) (*n* = 6, 6 mice). ImageJ software (National Institute of Health, Bethesda, MD, USA) was used to measure the percentage of the safranin‐O‐positive area and the thickness of the condylar cartilage.

### Immunofluorescence Staining

The immunofluorescence staining procedure was performed on paraffin sections following established laboratory protocols. After the sections were digested and repaired using a composite digestive solution and antigen repair solution (Boster, China), the sections were treated with 0.1% Triton X‐100 (Millipore Sigma, Burlington, MA, USA) for permeabilization, subsequently incubated with 5% goat serum (Beyotime) for blocking. These sections were incubated with primary antibodies at 4 °C, and Alexa Fluor‐labeled fluorescent secondary antibodies were processed on the next day. Tissue sections were counterstained with 4′,6‐diamidino‐2‐phenylindole (DAPI, Invitrogen, Waltham, USA) for nuclear visualization. Fluorescence images were acquired using a fluorescence microscope (FV1000, Olympus, Tokyo, Japan). Using Image J, quantitative analysis was performed by measuring the mean fluorescence intensity (MFI) from six randomly selected microscopic fields per experimental sample (*n* = 6, 6 mice). The antibodies used were Protein gene product 9.5 (PGP9.5, 1:400, ab8189, Abcam, Cambridge, UK), calcitonin gene‐related peptide 1 (CGRP, 1:300, sc‐57053, Santa Cruz Biotechnology, Santa Cruz, CA, USA), SLIT3 (1:300, DF9909, Affinity Biosciences, Cincinnati, OH, USA), TRAP‐α (1:300, sc‐373916, Santa Cruz Biotechnology), Osteocalcin (1:300, sc‐74495, Santa Cruz Biotechnology), β3‐tubulin (1:300, Cell Signaling Technology, Danvers, MA, USA) and CD51/61(1:300, AF5152, Affinity Biosciences, Cincinnati, OH, USA).

### Immunohistochemical Staining

Paraffin sections were digested and repaired routinely, and endogenous peroxidase activity was quenched by treating the sections with peroxidase blocking solution for 15 min. Subsequently, the sections were incubated with 5% goat serum (Beyotime) at room temperature for 1 h to block nonspecific binding, then primary antibody incubation was performed overnight at 4 °C. Next day, the sections were incubated with secondary antibodies. After PBS washing for three times, the sections were stained with diaminobenzidine and hematoxylin. The sections were then sealed using neutral gum. Semi‐quantitative analysis was performed using ImageJ software, where the percentage of the positively stained area relative to the total field of view was calculated (*n* = 6, 6 mice).^[^ ^66^ ^]^ The antibodies used were Matrix metalloproteinase (MMP9, 1:500, GTX100458, GeneTex, Irvine, CA, USA), MMP13 (1:50, GTX100665, GeneTex), Type‐II collagen (Col2, 1:50, ab34712, Abcam), SLIT3 (1:50, DF9909, Affinity Biosciences), and TRAP (1:50, sc‐373916, Santa Cruz Biotechnology).

### Tartrate‐resistant Acid Phosphatase Staining

Paraffin sections and mature osteoclasts were stained for TRAP, an enzymatic marker of osteoclasts. The TRAP stain kit (Solarbio, Beijing, China) was employed, and the staining was carried out in strict accordance with the manufacturer's instructions. The percentage of the positive area relative to the total field of view was quantified using ImageJ (*n* = 6, 6 mice).

### Flow Cytometric Analysis and Sorting

Six‐week‐old female SD rats were euthanized, and their mandibles were immediately dissected under sterile conditions. After soft tissue was removed and the mandibular joint incised, the bone samples were digested in 8 mL αMEM supplemented with 4.8 mg clostridial collagenase IA (Sigma, Switzerland). Samples were digested on an orbital shaker setting to 60 rpm for 4 h at 37° C. Thereafter, the digestion medium was collected, and bone pieces were thoroughly rinsed once with PBS. After centrifugation (450 g, 5 min) to obtain the cell pellet, the cell suspension was treated with ACK lysis buffer (Thermo Fisher Scientific) at room temperature for 3 min to remove red blood cells. Then, the lysis was terminated by adding culture medium. The single‐cell suspension was washed with PBS containing 2% FBS.^[^ ^67^ ^]^ The BMSCs were categorized into CD90‐positive (PE, M5/49.4.1, Elabscience, China), CD29‐positive (PE, E‐AB‐F1309D, Elabscience, China), CD34‐negative (PE, ab187284, Abcam, UK) and CD45‐negative (FITC, 30‐F11, Elabscience, China) populations. The osteoblasts were categorized into Runx2‐positive (FITC, sc‐390351, Santa Cruz Biotechnology, USA) populations. The osteoclasts were categorized into TRAP‐positive (PE, QA17A53, Biolegend, USA) populations. The BMMs were categorized into CD11b‐positive (PE, M1/70, Biolegend, USA) and CD68‐positive (PE, FA‐11, Biolegend, USA) populations. For the collection of sorted cells, 2 mL of TRIzol (#T9424, Sigma‐Aldrich) or complete DMEM was added to 15‐mL collection tubes. Analysis and sorting were performed using a flow cytometer (CytoFLEX, Beckman Coulter, Brea, CA, USA). Data analysis was performed using the Flow Jo 10.0 software (Flow Jo LLC, Ashland, OR, USA). Flow cytometry sorting was performed using the BD FACS Arialll system.

### Viral Anterograde Tracing of the TG

Mice were anesthetized and fixed on a stereotaxic apparatus. The scalp was incised to expose the anterior and posterior fontanelles, and the periosteal connective tissue on the surface of the skull was removed. After zeroing based on the anterior and posterior fontanelles, the skull was drilled at the positions of the two trigeminal ganglia (X = ±1.45, Y =  −1.34, Z = −5.65). A glass micropipette attached to a microsyringe (1 µL, Hamilton, USA) was inserted into the target site to deliver the recombinant AAV construct (rAAV‐CAG‐EGFP‐WPRE‐hGH polyA (supplied by BrainVTA, China; 2 × 10^12^ viral genomes mL^−1^) in a volume of 100 nL. After administration, the needle was left in place for 15 min before suturing the mice. One week later, the AAV9‐Slit3‐RNAi virus and control virus were intra‐articularly injected, followed by UAC model construction.^[^ ^68^, ^69^ ^]^

### Behavioral Tests

The mechanical hyperalgesia in the oral and maxillofacial regions of different groups of mice was assessed using calibrated Von Frey filaments (Ugo Basile, Italy) at 1 W, 3 W, and 6 W after UAC modeling (*n* = 6, 6 mice). Before testing, the mice were placed in a small cage with a limited space to prevent them from turning around and adapted to a quiet environment and suitable temperature (18–20 °C) for at least 1 h. After adaptation, the lowest Von Frey filament (0.008 g) was used to apply force to the skin area innervated by the mouse trigeminal nerve, with the filament bent at a 45° angle. The Von Frey filament was changed gradually till head withdrawal, and a positive reaction was recorded. The head withdrawal threshold was defined as the minimum force producing responses in 3 of 5 stimuli.^[^ ^70^ ^]^

The testing time nodes for the open field test (OFT) and the elevated plus maze test (EPM) were the same as Von Frey. Before testing, the mice were placed in the experimental environment to adapt for at least 1 h. Mouse spontaneous behavior and exploration were measured using OFT. The box was cleaned with 75% ethanol and wiped with a tissue. The mouse was recorded for 15 min once it was placed in the central area of the box. EPM was conducted to evaluate anxiety‐related exploratory behavior in mice. During the testing process, each mouse was gently placed on the central platform and allowed to explore freely for 5 min of testing. Behavioral parameters, including the time it takes to open and close the arm and the total distance traveled, were recorded.

### Preparation of Conditioned Media from BMMs and Osteoclasts

Primary bone marrow cells were obtained from the femur and tibia of 6‐week‐old wild‐type female mice, and the cells were cultured in alpha minimal essential medium (MEM). After 24‐h cultivation, nonadherent cells were cultured in a 24‐well plate at a density of 1×10^5^ cells per well. Following the adherence of BMMs to the wall, the conditioned medium was harvested. After centrifugation (699 g, 10 min, 4 °C), the conditioned media were aliquoted and stored at  −80 °C. Subsequently, the culture conditions were changed, and the BMMs were maintained in culture medium supplemented with 30 ng mL^−1^ macrophage colony‐stimulating factor (M‐CSF; R&D Systems Inc., USA) for more than 3 days, during which all cells became pre‐osteoclasts. By culturing BMMs with M‐CSF(15 ng mL^−1^) and receptor activator of nuclear factor kappa B ligand (RANKL,15 ng mL^−1^ R&D Systems Inc.) for more than 4 days, BMMs were completely differentiated into osteoclasts, and the culture medium was changed every 2 days. A commercial kit (G1492, Solarbio) was used to detect TRAP activity in mature osteoclasts. And after induction, the conditioned medium of mature osteoclasts was collected, centrifuged, and stored at −80 °C.

### Culture of TGs

The TG were dissected from the skull base of 3‐day‐old newborn mice. The tissue was minced and incubated in 0.1% collagenase (Millipore Sigma) at 37 °C and 5% CO_2_ for 20 min. Next, 0.25% trypsin (Thermo Fisher Scientific, USA) was added, and the mixture was incubated for 20 min. Then, alpha MEM medium containing 10% FBS was added to terminate the digestion process. After centrifuging the cell suspension (1000 rpm, 5 min, room temperature), the cell pellet was resuspended and laid on a pre‐coated poly‐L‐lysine (Millipore Sigma)‐coated culture plate at a density of ≈1.0 × 10^3^ cells per plate. TGs were maintained in a humidified atmosphere containing 5% CO_2_ at 37 °C in a neural basal culture medium supplemented with 0.5 mm L‐glutamine, 2% B‐27 (Invitrogen), and penicillin/streptomycin (1:100 dilution, Millipore Sigma). To evaluate the axonal morphology of the TGs in the different groups (SLIT3 protein, RPD353Mu01, Cloud‐clone, USA; Neutralizing antibodies against SLIT3, AF3629, R&D Systems, USA), immunofluorescence labeling (β3‐tubulin, 1:300, 2144, Cell Signaling Technology) and crystal violet staining (*n* = 6) were conducted.

### Real‐Time Quantitative Reverse Transcription PCR (qRT‐PCR)

Total RNA was extracted from mature osteoclasts and subchondral bone of rats using Trizol (Invitrogen), and its concentration and purity were measured (BioTek, Winooski, VT, USA). RNA was synthesized into cDNA using PrimeScript RT kit (Takara Bio, Japan), and qRT‐PCR was performed. The 2^−ΔΔCt^ method was used to estimate the fold changes relative to the control group.^[^ ^71^ ^]^ The primer sequences are shown in Table S1 (Supporting Information).

### Western Blotting

Mature osteoclast lysates or subchondral bone samples were collected and lysed. The supernatant was collected as the protein sample following centrifugation. After quantification using a bicinchoninic acid assay kit (E‐BC‐K318‐M, Elabscience, China), proteins in the sample were separated according to their molecular weight through sodium dodecyl sulfate‐polyacrylamide gel electrophoresis (SDS‐PAGE). The separated proteins were transferred from the gel onto a PVDF membrane (Bio‐Rad Laboratories, Hercules, USA). Then, the membrane was treated with a blocking buffer containing 5% BSA for 2 h, incubated with the first antibody overnight at 4 °C. After washing with TBST, the secondary antibody was added and incubated for 1 h. Enhanced Chemiluminescence (ECL) assay kit (Amersham Biosciences, Little Chalfont, UK) was used to detect immune reactive proteins. The antibodies used were anti‐mouse SLIT3 antibodies (1:2000, DF9909, Affinity Biosciences). For subchondral bone samples, the condyles of rats were frozen and crushed in liquid nitrogen before Western Blotting analysis.

### Enzyme‐Linked Immunosorbent Assay (ELISA)

Mature osteoclasts were washed with PBS, and the supernatant was replaced with 6 mL of serum‐free high glucose medium with phenol red for 1 day. Conditioned medium (CM) was obtained by filtration and stored at ‐80 °C. After protein concentration quantified, the SLIT3 concentration in the CM was measured using a commercially available ELISA kit (JM‐13297M1, Jingmei, Jiangsu, China). The detection range was 1.0–240 ng mL^−1^, with a minimum detectable dose of less than 1.0 ng mL^−1^. Mean values of duplicate samples were reported. For subchondral bone samples, the rat condyles were frozen in liquid nitrogen, crushed, and other procedures were as same as before.

### Micro‐Computed Tomography (Micro‐CT)

Micro‐computed tomography analysis was performed using an Inveon µCT scanner (Siemens AG, Germany). The acquired projection images were reconstructed into 3D volumes using the manufacturer's proprietary software (Inveon Research Workplace, Siemens Medical Solutions USA, Inc., USA). For quantitative analysis of trabecular microarchitecture and bone mineral density, a 0.25 × 0.25 × 0.25 mm^3^ cubic region of interest (ROI) was selected from the central subchondral region of the condyle. Quantitative microstructural analysis included the following parameters: morphometric dimensions (width, length), Bone structural indices (BS/BV, BV/TV), Bone mineral density (BMD), and Trabecular microarchitecture parameters (Tb.N, Tb.Th, Tb.Sp) (*n* = 6, 6 mice).^[^ ^66^ ^]^

### Statistical Analyses

All experimental procedures and quantitative evaluations were performed under blinded conditions. Results are expressed as means ± standard deviations; the sample size (*n*) for each statistical analysis is 6. Before conducting parametric analyses, the fundamental assumptions of normality and homoscedasticity were systematically evaluated using appropriate statistical tests. Comparisons were analyzed using Student's *t*‐test and one‐ or two‐way analysis of variance (ANOVA) followed by the Holm–Šidák multiple comparison test. Statistical analyses were performed using GraphPad Prism 9 software (GraphPad Software, La Jolla, USA). Statistical significance was defined at α = 0.05.

## Conflict of Interest

The authors declare that they have no conflict of interest.

## Author Contributions

W.Z., W.Q., and J.G. contributed equally to this work. K.J. and J.Y. conceived and designed the project. W.Z. performed the experiments. W.Q. and X.H. contributed to data interpretation. Y.G., Z.M., X.Z. contributed to animal experiments. J.H., J.G., and J.L. analyzed the data. K.J., J.Y., L.N., B.G., and C.L. wrote and polished the manuscript.

## Supporting information



Supporting Information

## Data Availability

The data that support the findings of this study are available in the supplementary material of this article.
