# Supplementary material for: Osteoclast‐Derived SLIT3 Mediates Osteoarthritis Pain and Degenerative Changes
Source: Adv Sci (Weinh). 2025 Nov 19;13(7):e17545. doi: 10.1002/advs.202517545 (PMC12866685; doi:10.1002/advs.202517545)
Supplement: Supplementary file 1 — Supporting Information [file ADVS-13-e17545-s001.docx]

**Supporting Information**

**Osteoclast-Derived SLIT3 Mediates Osteoarthritis Pain and Degenerative Changes**

*Weiwei Zhu^#^, Wenpin Qin^#^, Jialu Gao^#^, Yihan Guo, Xiaoxiao Han, Zhangyu Ma, Xiaokang Zhang, Jie He, Jing Liu, Bo Gao, Changjun Li, Lina Niu, Jianfei Yan** *and Kai Jiao**

*Co-first Author(s)*^#^*, and Corresponding Author(s*)*

*
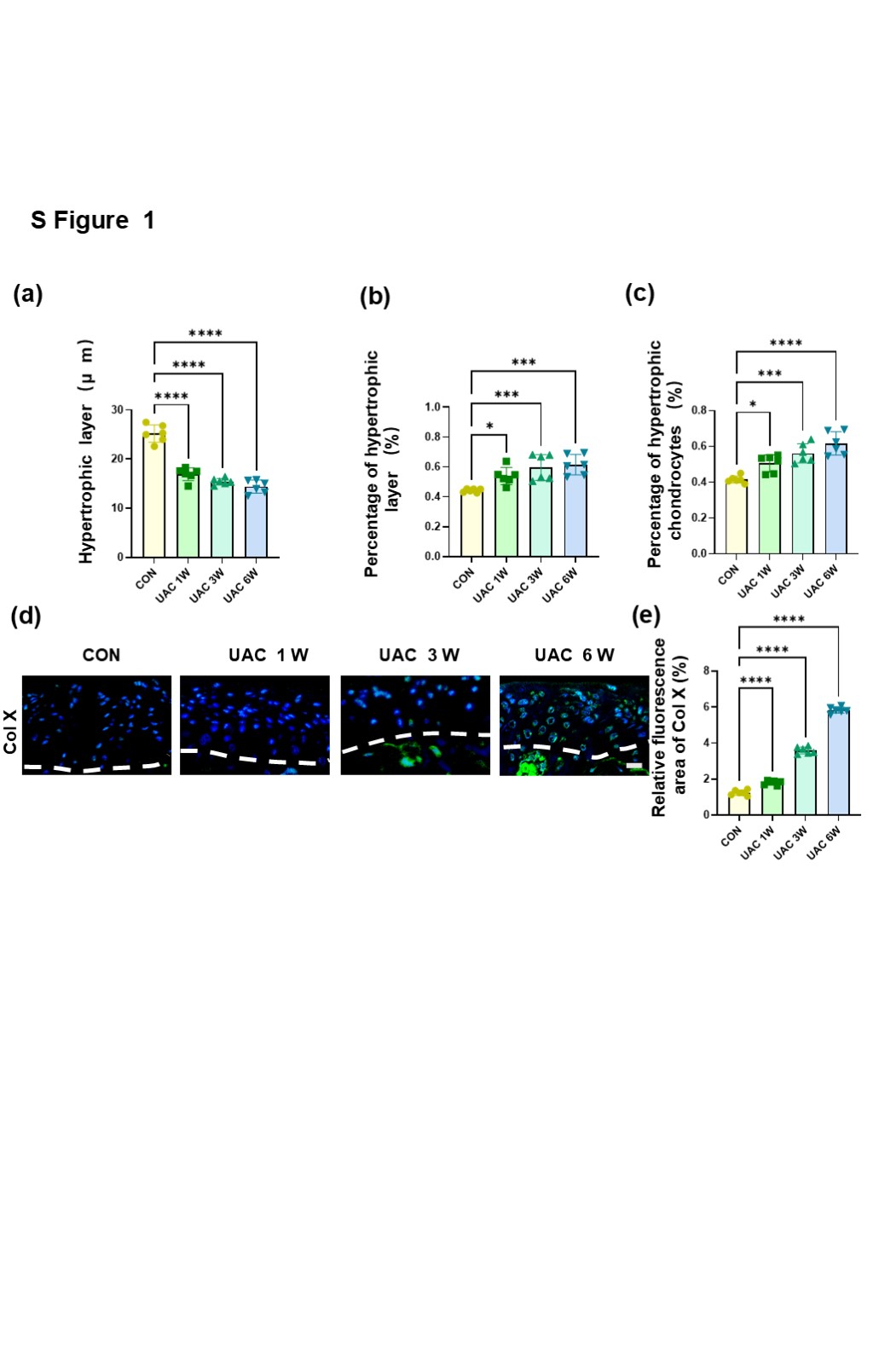
*

**Supplemental Figure S1. (a-c)** Quantitative analysis of the thickness of the hypertrophic layer, the percentage of hypertrophic layer, the percentage of hypertrophic chondrocytes. *n* = 6. **(d)** Representative images of immunofluorescence staining for Col-X in the mandibular condyle. Scale bars: 10 µm. (**e)** Quantitative analysis in panel **(d).** *n* = 6. Statistical analyses were performed using one-way ANOVA with Holm-Šidák multiple comparison tests. **p* < 0.05. ****p* < 0.001. *****p* < 0.0001.


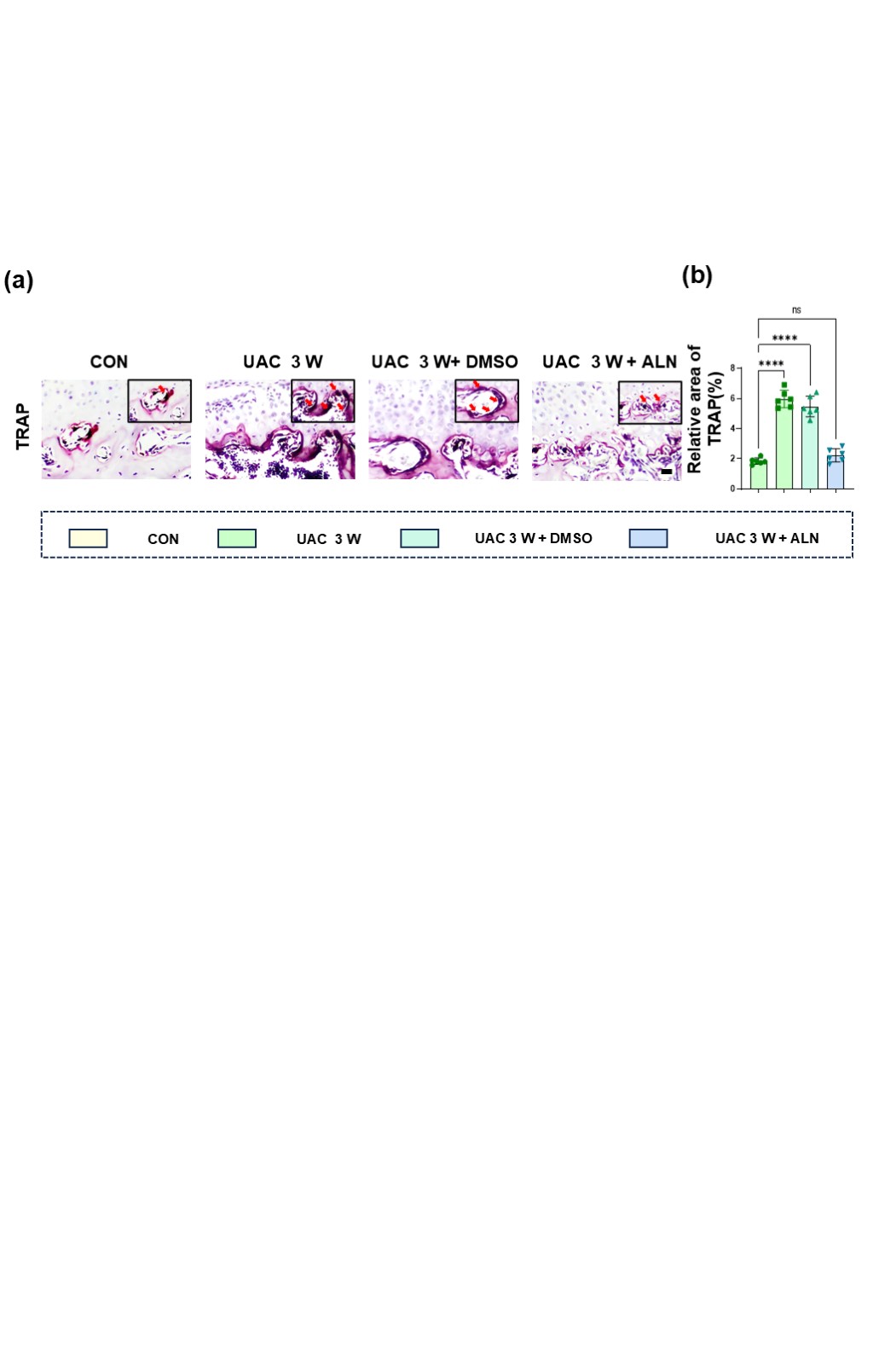


**Supplemental Figure S2. (a)** Representative images of TRAP staining in subchondral bone marrow. Red arrow means the TRAP^+^ cells. Scale bars:70µm. **(b)** Quantitative analysis of relative area of TRAP^+^ osteoclasts in subchondral bone marrow. *n* = 6. Statistical analyses were performed using one-way ANOVA with Holm-Šidák multiple comparison tests. *****p*<0.0001. *ns*: no significance.

**
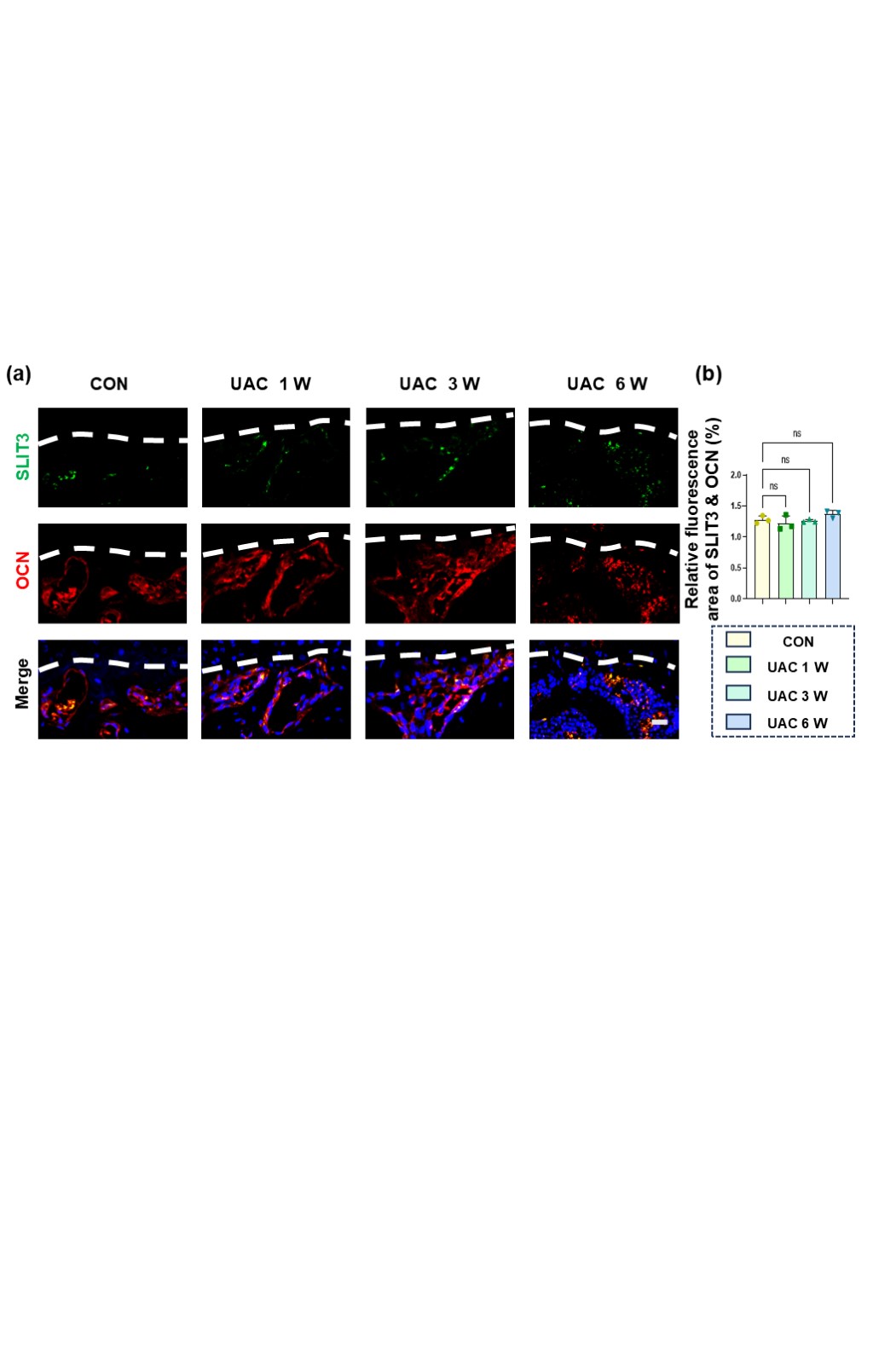
**

**Supplemental Figure S3.** **(a)** Representative images of SLIT3 (green) and osteocalcin (red) co-stained cells in subchondral bone in the CON and UAC groups. Scale bars: 10 µm. **(b)** Quantitative analysis in panel **(a)**. *n* = 6. Statistical analyses were performed using one-way ANOVA with Holm-Šidák multiple comparison tests. *ns*: no significance.

**
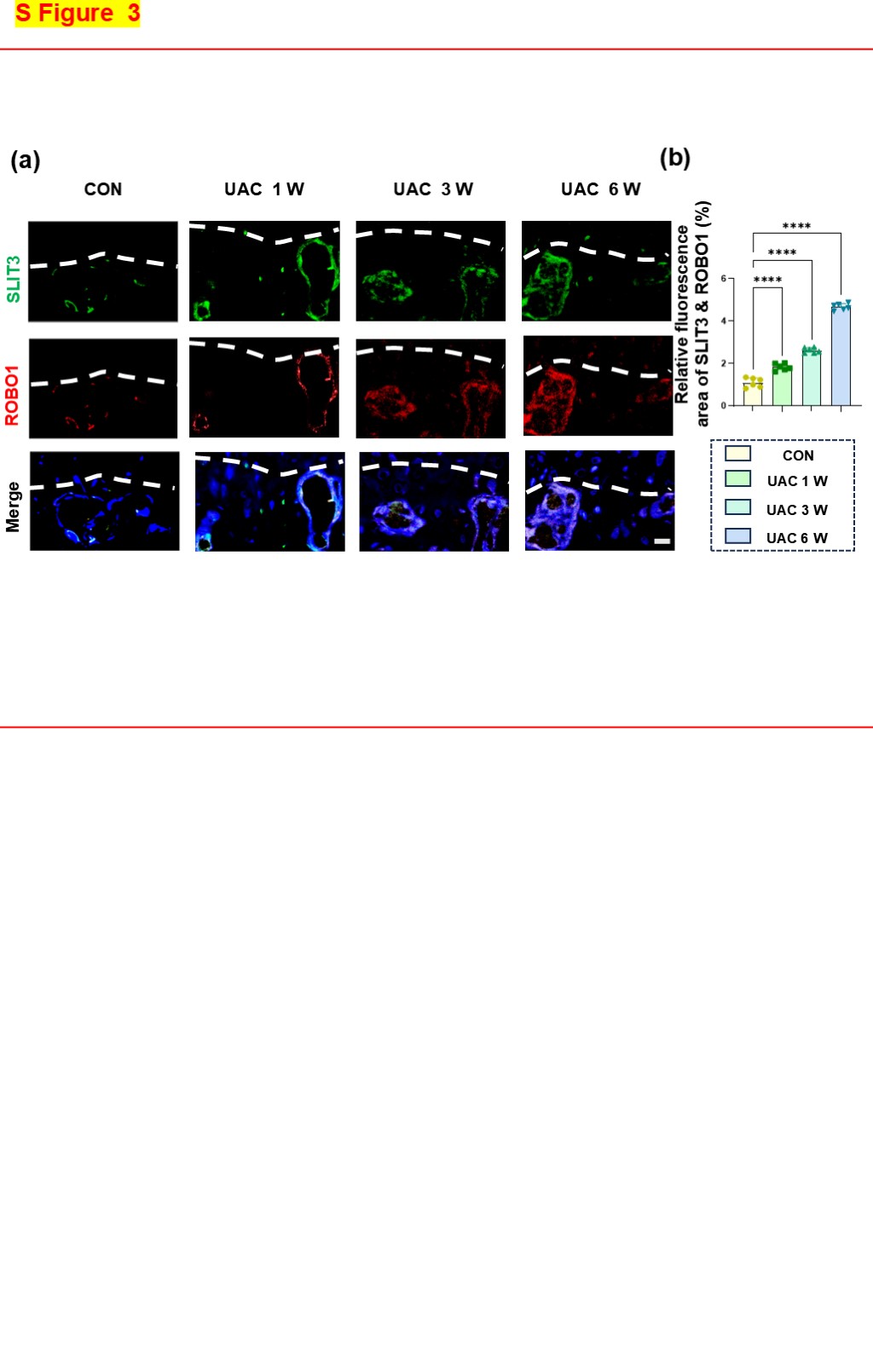
 Supplemental Figure S4.** **(a)** Representative images of SLIT3 (green) and ROBO1 (red) co-stained cells in subchondral bone in the CON and UAC groups. Scale bars: 10µm. **(b)** Quantitative analysis in panel **(a)**. *n* = 6. Statistical analyses were performed using one-way ANOVA with Holm-Šidák multiple comparison tests. *****p*<0.0001.


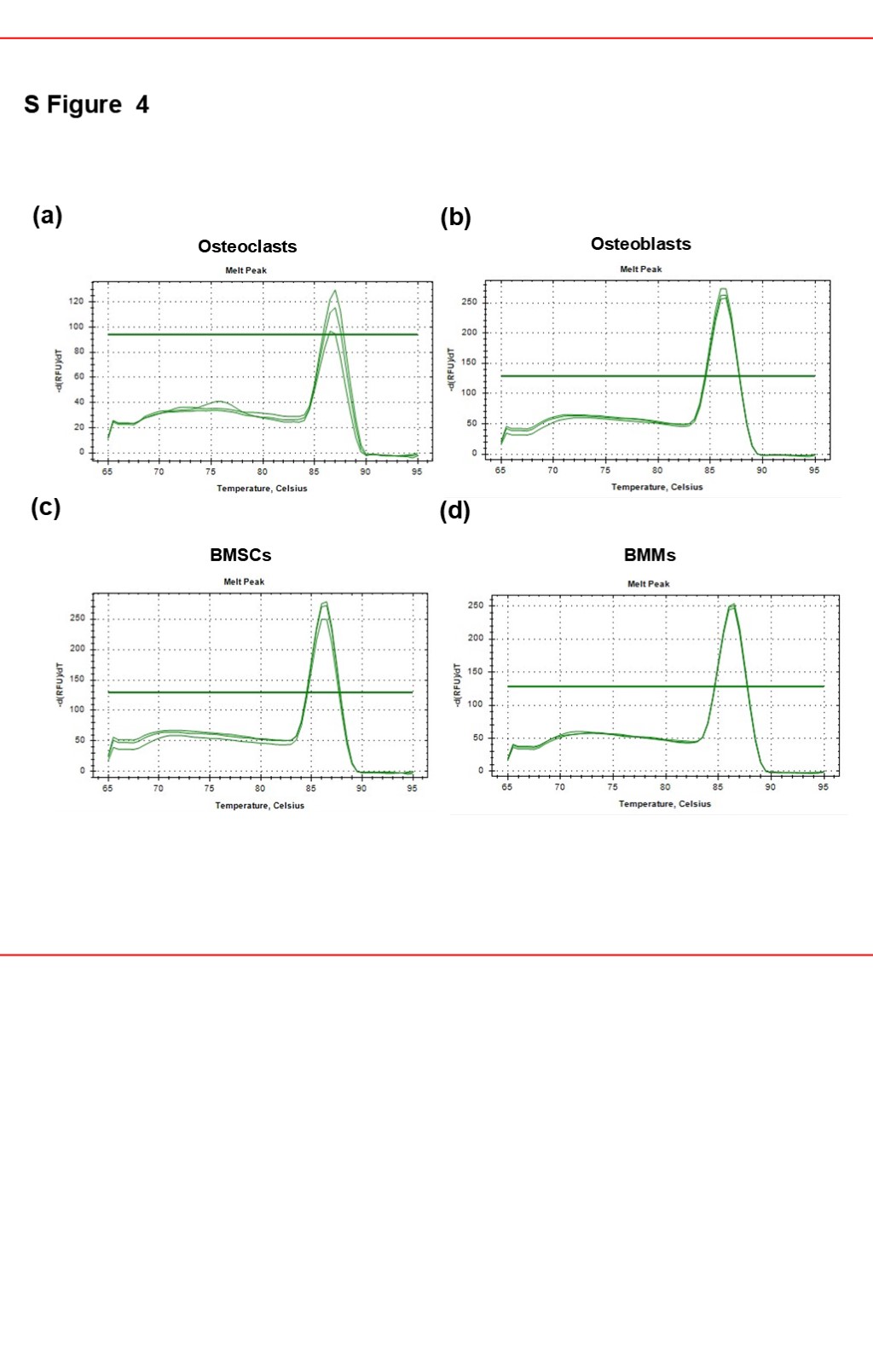


**Supplemental Figure S5.** RT-qPCR melting curve of *Slit3* expression in osteoclasts, osteoblasts, BMSCs, and BMMs.
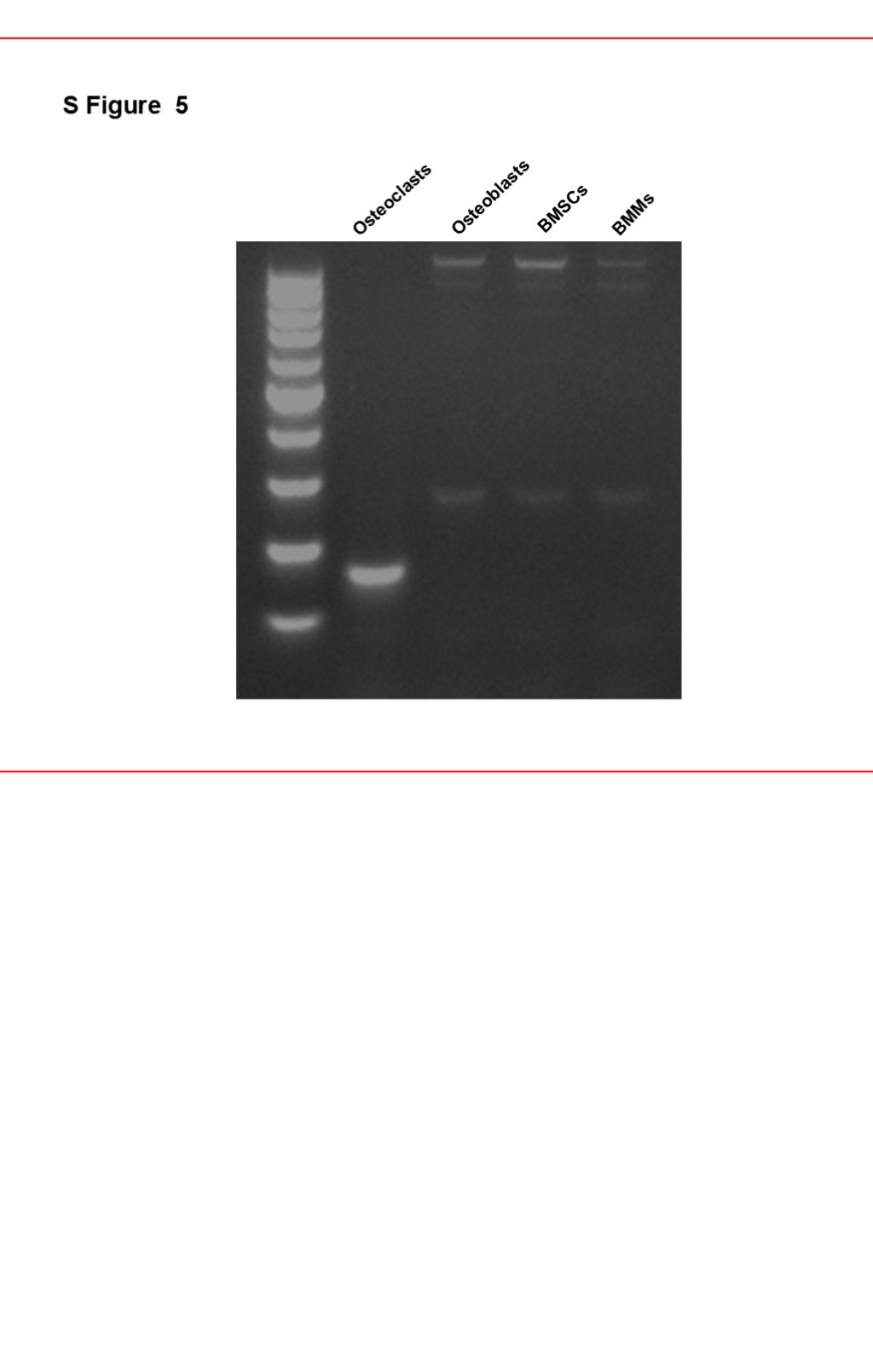


**Supplemental Figure S6.** Representative agarose gel electrophoresis of specific amplification band (145 bp) for *Slit3* from osteoclasts, osteoblasts, BMSCs, and BMMs.


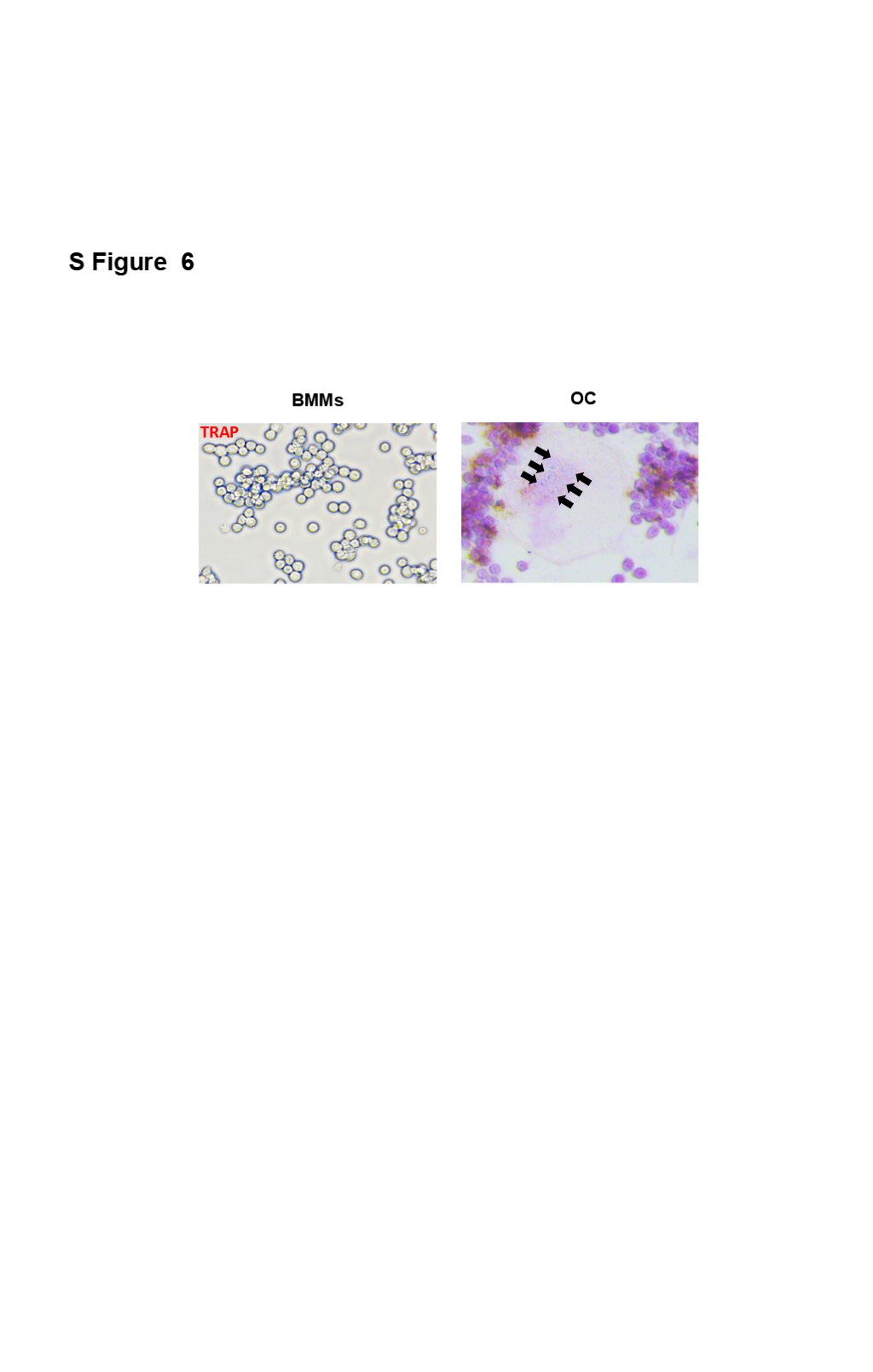


**Supplemental Figure S7.** Representative images of bone marrow-derived macrophages (BMMs) and osteoclasts (OC).


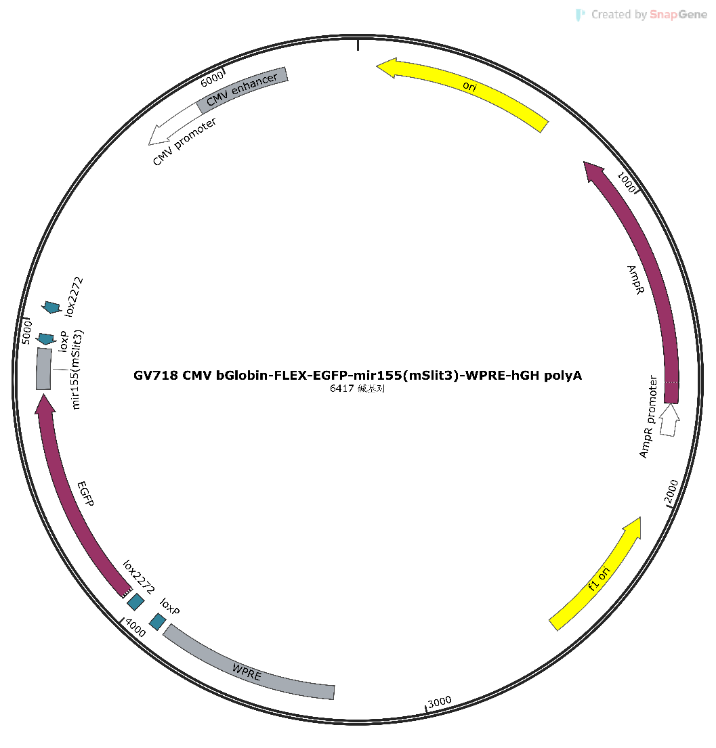


**Supplemental Figure S8.** The vector map for inserting target sequences. The viral vector we employed targets Slit3 (NM_011412, Mouse) and utilizes the GV718 backbone: CMV bGlobin-FLEX-EGFP-MIR155(mSlit3)-WPRE-hGH polyA. This viral vector was administered through intra-articular injection into TRAP-Cre mice.


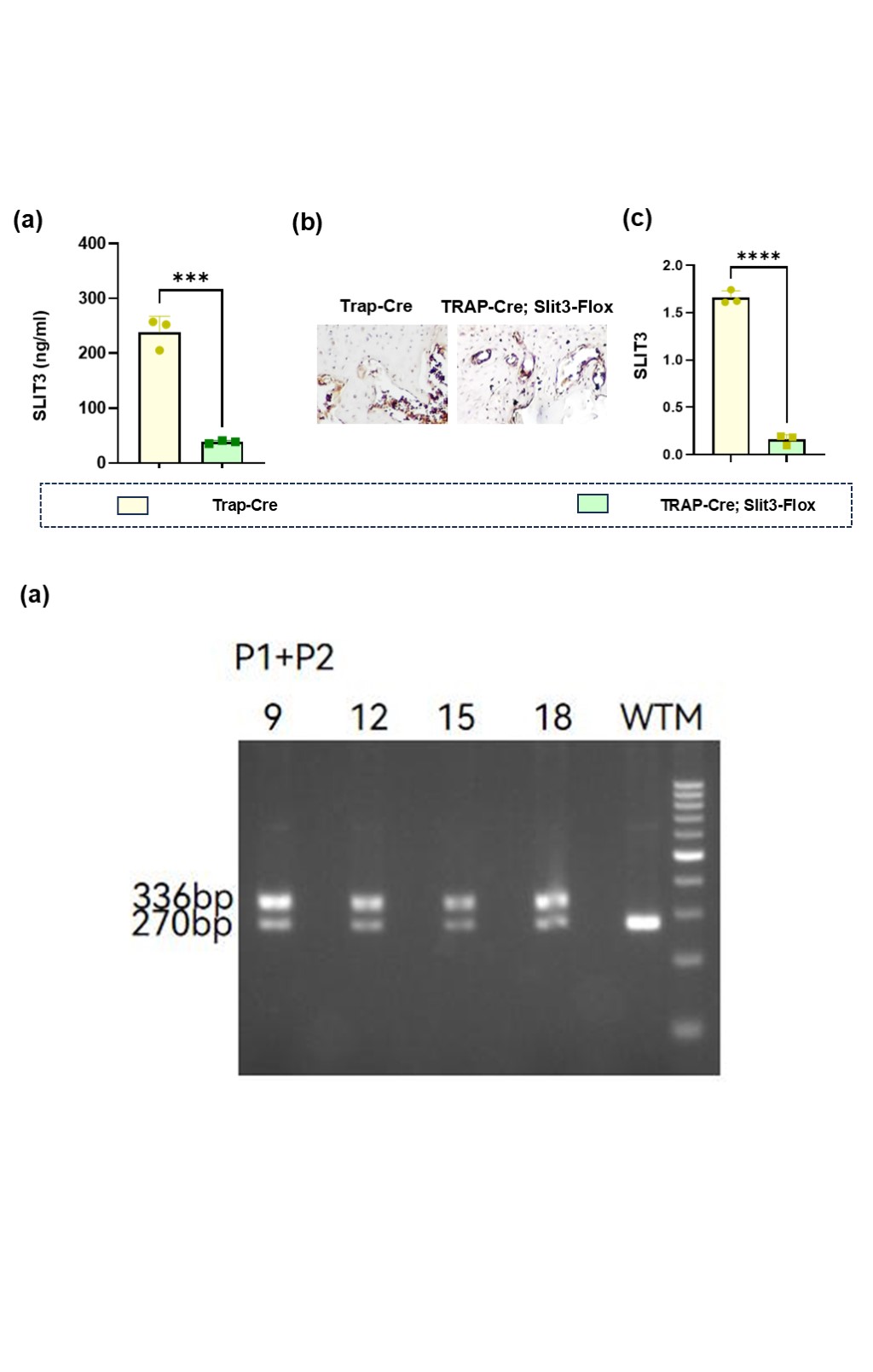


**Supplemental Figure S9.** After extracting the genomic DNA of SLIT3-Flox mice (Modelorg, China), gene amplification was performed by polymerase chain reaction (PCR) using the following primers: P1(AGGCACCAGGGAGAGAGTAT) and P2 CTGAACGGAGTCCTGTGTCTC, then DNA gel electrophoresis was performed. Wild type: P1P2=270 bp; Heterozygote: P1P2=270 bp and 336 bp; Homozygote: P1P2=336 bp. WT: Wild type. Marker: 100bp DNA marker from Transgen.


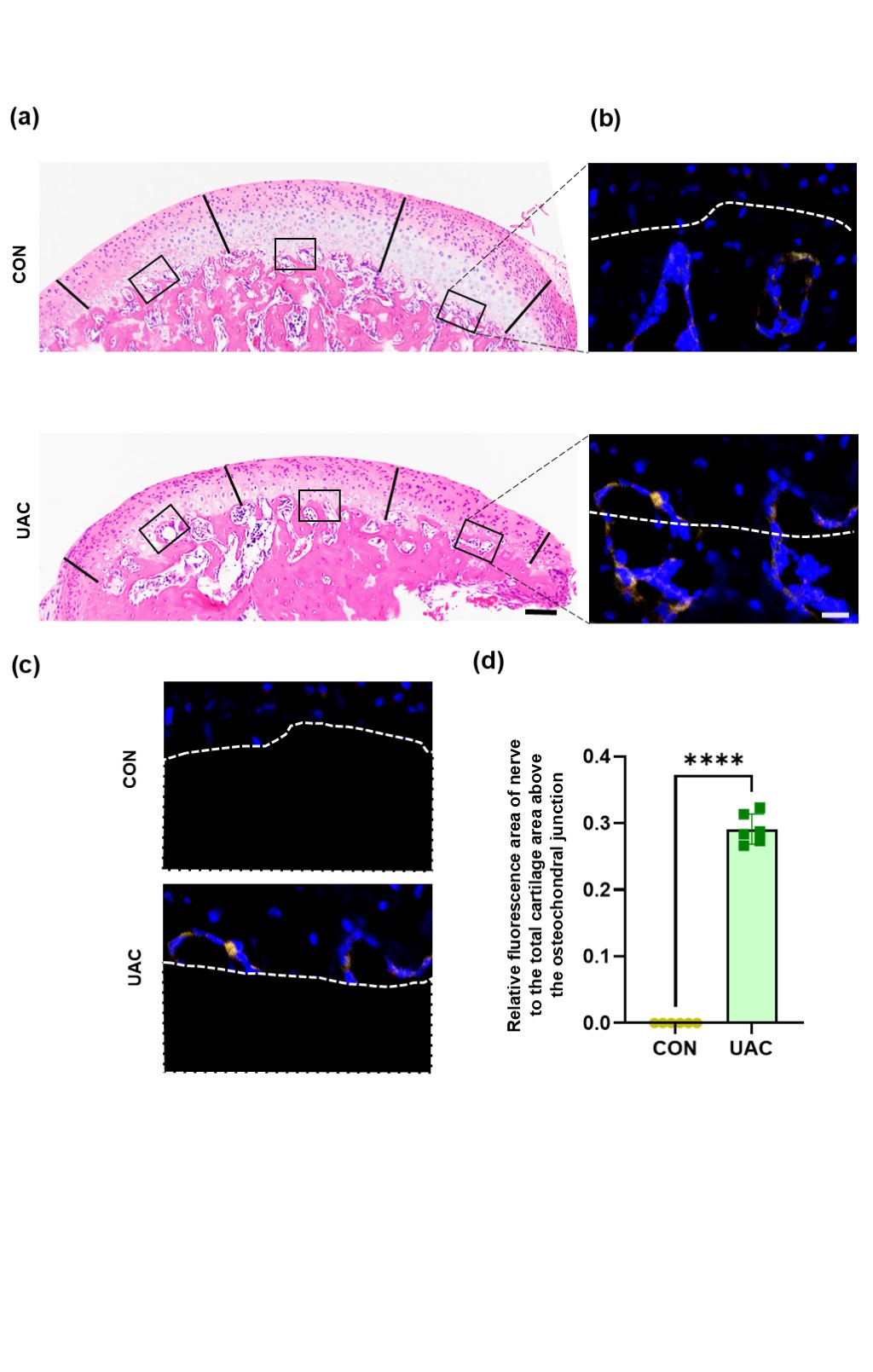


**Supplemental Figure S10.** **(a)** A typical central sagittal section of TMJ stained with HE. The bold vertical lines divide the articular cartilage of mandibular condyle into three equal sections (anterior, middle, and posterior). The boxes depict regions of interest in each section. Scale bars: 200 µm. n = 6. **(b)** Representative images of PGP9.5 (red) and CGRP (green) co-stained cells along the TMJ osteochondral junction in the CON and UAC groups. Scale bars: 20 µm. n = 6. **(c)** Representative images of PGP9.5 (red) and CGRP (green) co-stained cells showing only above the osteochondral junction. Scale bars: 20 µm. n = 6. **(d)** Quantitative analysis of sensory nerve-positive area to the total cartilage area above the osteochondral junction. Statistical analyses were performed using Student’s t-test. ****p < 0.0001.

**Supplemental Table S1. Primer sequences used for ​​quantitative real-time polymerase chain reaction.** *Slit3*, Slit Homolog 3; *CGRP*, Calcitonin-gene-related peptide; *Ngfr*, Nerve Growth Factor Receptor; *Ntrk1*, Neurotrophic Receptor Tyrosine Kinase 1; *GAPDH*, Glyceral dehyde-3-phosphate dehydrogenase; F, forward primer; R, reverse primer;

| **Primer** | **Sequences** |
| --- | --- |
| M-Slit3-F | 5’-AGACCCTGAACCTGGTGGTAGAC-3’ |
| M-Slit3-R | 5’-TCTGCACCCTGGCGTAAGG-3’ |
| R-Slit3-F | 5’-GAGGGCTATGAGGGACCTTTGT-3’ |
| R-Slit3-R | 5’-GCGGATGGCTTCACGGAC-3’ |
| R- netrin 3-F | 5’-CAGAGCATTAGCCTGCTGGA-3’ |
| R- netrin 3-R | 5’-AGTTTCAAGCGGGTATGGGG-3’ |
| R-netrin 4-F | 5’-ACGAGGCAAGAGAACACTCTATCC-3’ |
| R-netrin 4-R | 5’-CCTCATAGCCAGCTACCAGGTACT-3’ |
| R-CGRP-F | 5’-TCCAGGCAGTTCCTTTGAGG-3’ |
| R-CGRP-R | 5’-GCCAGTAGGCGAGCTTCTTC-3’ |
| R-Ngfr-F | 5’-ACCTCATTCCTGTCTATTGCTCC-3’ |
| R-Ngfr-R | 5’-GCGCCTTGTTTATTTTGTTTGC-3’ |
| R-Ntrk1-F | 5’-GCAGGGACATCTACAGCACA-3’ |
| R-Ntrk1-R | 5’-CCGAAGCTCCACACATCACT-3’ |
| R-GAPDH-F | 5’-GGCACAGTCAAGGCTGAGAATG-3’ |
| R-GAPDH-R | 5’-ATGGTGGTGAAGACGCCAGTA-3’ |
| M-GAPDH-F | 5’-TGTGTCCGTCGTGGATCTGA-3’ |
| M-GAPDH-R | 5’-TTGCTGTTGAAGTCGCAGGAG-3’ |

**Supplemental Table S2. Detailed cycle threshold (Ct) values of *Slit3*.** The primary culture wells in the six-well plate were labeled as 1, 2, and 3, and their respective PCR replicates were designated as 1-1, 1-2, and 1-3 during sample loading.

| **Cell type** | **Value** | | |
| --- | --- | --- | --- |
| BMMs | 1 | 1-1 | 36.33 |
|  |  | 1-2 | 36.27 |
|  |  | 1-3 | 36.47 |
|  | 2 | 2-1 | 36.74 |
|  |  | 2-2 | 36.33 |
|  |  | 2-3 | 36.38 |
|  | 3 | 3-1 | 36.56 |
|  |  | 3-2 | 36.61 |
|  |  | 3-3 | 36.32 |
| Osteoclasts | 1 | 1-1 | 34.56 |
|  |  | 1-2 | 34.50 |
|  |  | 1-3 | 34.22 |
|  | 2 | 2-1 | 34.27 |
|  |  | 2-2 | 34.58 |
|  |  | 2-3 | 34.36 |
|  | 3 | 3-1 | 34.31 |
|  |  | 3-2 | 34.54 |
|  |  | 3-3 | 34.46 |
| Osteoblasts | 1 | 1-1 | 36.24 |
|  |  | 1-2 | 36.09 |
|  |  | 1-3 | 36.31 |
|  | 2 | 2-1 | 36.40 |
|  |  | 2-2 | 35.91 |
|  |  | 2-3 | 36.04 |
|  | 3 | 3-1 | 36.08 |
|  |  | 3-2 | 36.33 |
|  |  | 3-3 | 36.28 |
| BMSCs | 1 | 1-1 | 36.49 |
|  |  | 1-2 | 36.30 |
|  |  | 1-3 | 36.22 |
|  | 2 | 2-1 | 36.74 |
|  |  | 2-2 | 36.34 |
|  |  | 2-3 | 36.46 |
|  | 3 | 3-1 | 36.37 |
|  |  | 3-2 | 36.76 |
|  |  | 3-3 | 36.48 |
